# Natural killer cell receptor signaling and activation depend on cell cycle stages

**DOI:** 10.1371/journal.pcbi.1014825

**Published:** 2026-10-13

**Authors:** Darren Wethington, Indrani Nayak, Helle Jensen, William C. Stewart, Oscar A. Aguilar, Shih-Yu Chen, Garry P. Nolan, Gregory K. Behbehani, Lewis L. Lanier, Jayajit Das

**Affiliations:** 1 Steve and Cindy Rasmussen Institute for Genomic Medicine, Abigail Wexner Research Institute at Nationwide Children’s Hospital, Columbus, Ohio, United States of America; 2 Biomedical Sciences Graduate Program, The Ohio State University, Columbus, Ohio, United States of America; 3 Department of Microbiology and Immunology and the Parker Institute for Cancer Immunotherapy, University of California-San Francisco, San Francisco, California, United States of America; 4 GIG Statistical Consulting, LLC, Columbus, Ohio, United States of America; 5 Institute of Biomedical Sciences, Academia Sinica, Taipei, Taiwan; 6 Department of Pathology, Stanford University School of Medicine, Palo Alto, California, United States of America; 7 Division of Hematology, The Ohio State University, Columbus, Ohio, United States of America; 8 Pelotonia Institute for Immuno-Oncology, The James Comprehensive Cancer Center, The Ohio State University, Columbus, Ohio, United States of America; 9 Department of Pediatrics, College of Medicine, The Ohio State University, Columbus, Ohio, United States of America; 10 Biophysics Program, The Ohio State University, Columbus, Ohio, United States of America; University of California San Diego Division of Biological Sciences, UNITED STATES OF AMERICA

## Abstract

Natural Killer (NK) cells are lymphocytes of the innate immunity and provide key protection against infection and cancer. NK cells express a wide array of inhibitory and activating receptors on the cell surface including the activating receptor NKG2D. Receptor signaling in NK cells leads to rapid post-translational modification (e.g., phosphorylation) of sub-cellular signaling proteins within minutes of receptor stimulation that ultimately give rise to diverse effector functions including cell proliferation. Recent single-cell mass cytometry (i.e., CyTOF) experiments in macrophages showed variations of abundances of phosphorylated signaling proteins across cell cycle stages indicating a dependence of cell signaling kinetics on an order of magnitude slower kinetics (~ several hours) of cell cycle transitions. We investigated cell cycle dependence of NKG2D signaling kinetics in NK cells using longitudinal CyTOF measurements up to 256 minutes post stimulation performed on IL-2-treated NKG2D-stimulated primary human CD56^dim^ NK cells. The CyTOF experiments revealed cell stage specific kinetics of signaling proteins such as pVav1, pErk, and pS6, with the degranulation marker CD107a showing its largest upregulation specifically in the combined G2 and M (G2 - M) stage. We developed stochastic minimal computational models coupling signaling and cell cycle processes to investigate the effect of cell cycle transitions on NK cell receptor induced signaling kinetics. These models suggest that the observed cell stage specific NK cell receptor signaling kinetics arise due to active modulation of the receptor signaling kinetics by cell cycle progression. The framework can be extended to study the interplay between receptor signaling and cell cycle progression in other cell types.

## Author summary

NK cells are innate lymphocytes that protect against infection and cancer. They express receptors such as NKG2D, which enable them to sense their environment and respond to healthy or diseased cells. Upon binding to cognate ligands, receptors such as NKG2D induce a series of rapid biochemical signaling reactions within minutes, regulating NK cell effector functions such as cell proliferation or cytokine release. In contrast, dividing NK cells progress through the cell cycle (G1 → S → G2 → M) on a much slower timescale of hours. Since protein synthesis, including that of signaling components, occurs during cell cycle progression, receptor signaling kinetics are likely to depend on cell cycle stage. We performed single-cell CyTOF experiments on IL-2-treated primary human NK cells stimulated with anti-NKG2D antibodies, measuring signaling and cell cycle-associated proteins over time. The results revealed cell-stage-specific signaling kinetics, including early activation and degranulation responses. We developed stochastic minimal models that couple receptor signaling with cell cycle transitions to quantify how cell cycle progression regulates signaling kinetics. Analysis of CyTOF data supports an active role of the cell cycle in modulating NKG2D signaling. This framework can be extended in other cell types.

## Introduction

Natural Killer (NK) cells are lymphocytes of our innate immune system and provide important protection against infection and tumors [1–3]. NK cells interact with target cells via a wide array of plasma membrane-bound activating and inhibitory NK cell receptors (NKRs), co-receptors, adhesion, and cytokine receptors [2,3]. Binding of these receptors expressed on the surface of NK cells initiate subcellular biochemical signaling reactions within seconds to minutes [4–6], which may eventually unleash NK cell effector functions [1,2] ranging from cell proliferation, lysis of target cells, and secretion of cytokines such as interferon gamma. NK cell division can take many hours [7,8] to fully manifest, which is several orders of magnitude longer than the time scales (~ seconds to minutes) of biochemical signaling reactions.

Proliferating eukaryotic cells spend most of the cell cycle in interphase, during which DNA, proteins, and metabolites are synthesized in preparation for division. Interphase comprises the G1, S, and G2 stages (G1 → S → G2), followed by mitosis (M) and cytokinesis, which produces two daughter cells in G1 that either continue through the cell cycle or exit the cell cycle to enter a quiescence state G0 [9]. As single NK cells progress through cell cycle transitions, many signaling proteins such as kinases and phosphatases, as well as their substrate proteins, are synthesized to maintain a stable copy number/per cell or abundance during cell growth [10,11]. Consequently, when receptors on NK cells at different cell cycle stages (e.g., G1, S) are stimulated by cytokines or ligands expressed on target cells, their signaling kinetics can differ across cell cycle stages. To illustrate, the abundance (or number of molecules per cell) of a phosphorylated form of a signaling protein (e.g., pVav1) can vary in NK cells at different cell cycle stages as the abundances of phosphorylating and dephosphorylating enzymes, as well as the abundance of the substrate protein (e.g., Vav1) change throughout the cell cycle. In addition, proteins regulating cell cycle processes, such as cyclindependent kinases (CDKs), whose abundances vary across the cell cycle, can actively participate in chemically modifying signaling proteins. For example, in HeLa cells the phosphorylation of the site S473 in signaling protein Akt is regulated by CDK2 [12]. This produces variations in the abundances of the phosphorylated protein Akt-pS473 similar to that of CDK2 abundances across cell cycle stages while the total Akt abundance remains unchanged [12]. In another example, CDK proteins in G2-M stage phosphorylate Wnt-binding co-receptor LRP6 promoting Wnt signaling in the stage [13]. The dependence of NK cell signaling on cell cycle stages is not well characterized.

Here, we investigated the cell cycle dependence on NK cell signaling and activation by performing cytometry by time-of-flight (CyTOF) measurements on IL-2-treated primary human CD56^dim^ NK cells stimulated by plate-bound anti-NKG2D antibodies at different time points. A total of 40 different proteins were evaluated by CyTOF, mostly pertaining to signaling proteins, surface functional markers (e.g., CD107a) and cell cycle markers. The kinetics of the average abundance of several signaling proteins (e.g., pVav1, pErk, pAkt, pS6) and surface marker proteins (e.g., CD69, CD107a) post-NKG2D stimulation showed differences across cell cycle stages. The mechanisms underlying such cell cycle stage-dependent variations in the abundance of signaling products can involve changes in biochemical reaction propensities generated by cell cycle-dependent variations in reactant protein concentrations and/or the active involvement of CDKs in altering the rates of chemical modifications in signaling products. We developed minimal mathematical and computational stochastic models that couple cell cycle transitions and signaling reactions to investigate different hypotheses (or mechanisms) regarding the role of cell cycle stages in producing variations in signaling protein abundances in NK cells. Application of the models to describe the CyTOF data supported the hypothesis that cell cycle processes actively influence signaling reactions producing pAkt and pS6. The developed framework, which integrates modeling and CyTOF experiments, provides a path forward for uncovering mechanisms underlying the regulation of cell signaling by cell cycle transitions.

## Results

### Phosphoprotein intensities show cell cycle stage specific kinetics in NKG2D-stimulated IL-2-treated primary NK cells

The abundances of proteins involved in NKG2D signaling may vary across cell cycle stages in NK cells stimulated with NKG2D ligands. This variation could arise from differences in the concentrations of interacting proteins in cells at distinct cell cycle stages, as well as from regulation of signaling kinetics by cell cycle-associated proteins (e.g., CDKs) that drive cell cycle progression (Fig 1). We investigated the variation of abundances of NKG2D signaling proteins in primary human NK cells obtained from a healthy donor. Primary human CD56^dim^ NK cells were treated with IL-2 for 24 hours to prime the NKG2D signaling pathway and then stimulated with plate-bound anti-NKG2D agonist antibodies. NKG2D transmits signals through the DAP10 adaptor protein, which recruits and activates the phosphatidylinositol 3-kinase [14]. The NK cells were profiled at 2, 4, 8, 16, 32, 64, and 256 minutes post-NKG2D stimulation using CyTOF [15], which assayed 31 different signaling proteins and surface markers simultaneously (Fig 2A). The various cell cycle stages (i.e., G0, G1, S, and G2 - M) were gated using UMAP based on the cell cycle markers, 5-iodo-2-deoxyuridine (IdU), cyclin B1, phosphorylated histone 3 (pH3), phosphorylated retinoblastoma protein (pRb) and Kiel 67 (Ki67) (Fig 2B, S1 Fig, and more details in **Materials and Methods**). The analysis showed ~10–20% of the NK cells were in G0, ~ 60–70% were in G1, and ~10% were in S and G2 + M stages, respectively, throughout the various time points post-NKG2D stimulation (see Fig 2C and 2D). The data show cell cycle stage-specific changes in protein intensities across time points post-NKG2D stimulation (Fig 2E). To systematically characterize these stage-specific protein kinetics, we quantified the geometric mean of protein intensities in G1, S, and G2 - M across the NKG2D stimulation time course, enabling direct comparisons of signaling kinetics between cell cycle stages (Fig 2F, S2 Fig).This analysis revealed that mean intensities of several proteins, including CD69, pS6, pVav1, pAkt, pCrkL, and pPLCγ2, displayed uneven temporal kinetics within each cell cycle stage. In addition, several signaling and functional markers including pS6, pAkt, pErk1/2, pCrkL, pPLCγ2, pSLP76, and CD69 consistently reached their highest mean intensities in the G2 - M phase across multiple time points following NKG2D stimulation. The protein intensities at t = 256 mins are likely to be influenced by the transitions of cells from the prior cell cycle stage as the NKG2D treatment times (256 min) is comparable to the duration of some cell cycle stages. These results highlight clear differences in signaling kinetics across cell cycle stages and provide a more detailed view of cell cycle stage-dependent responses during NK cell activation.

**Fig 1:**
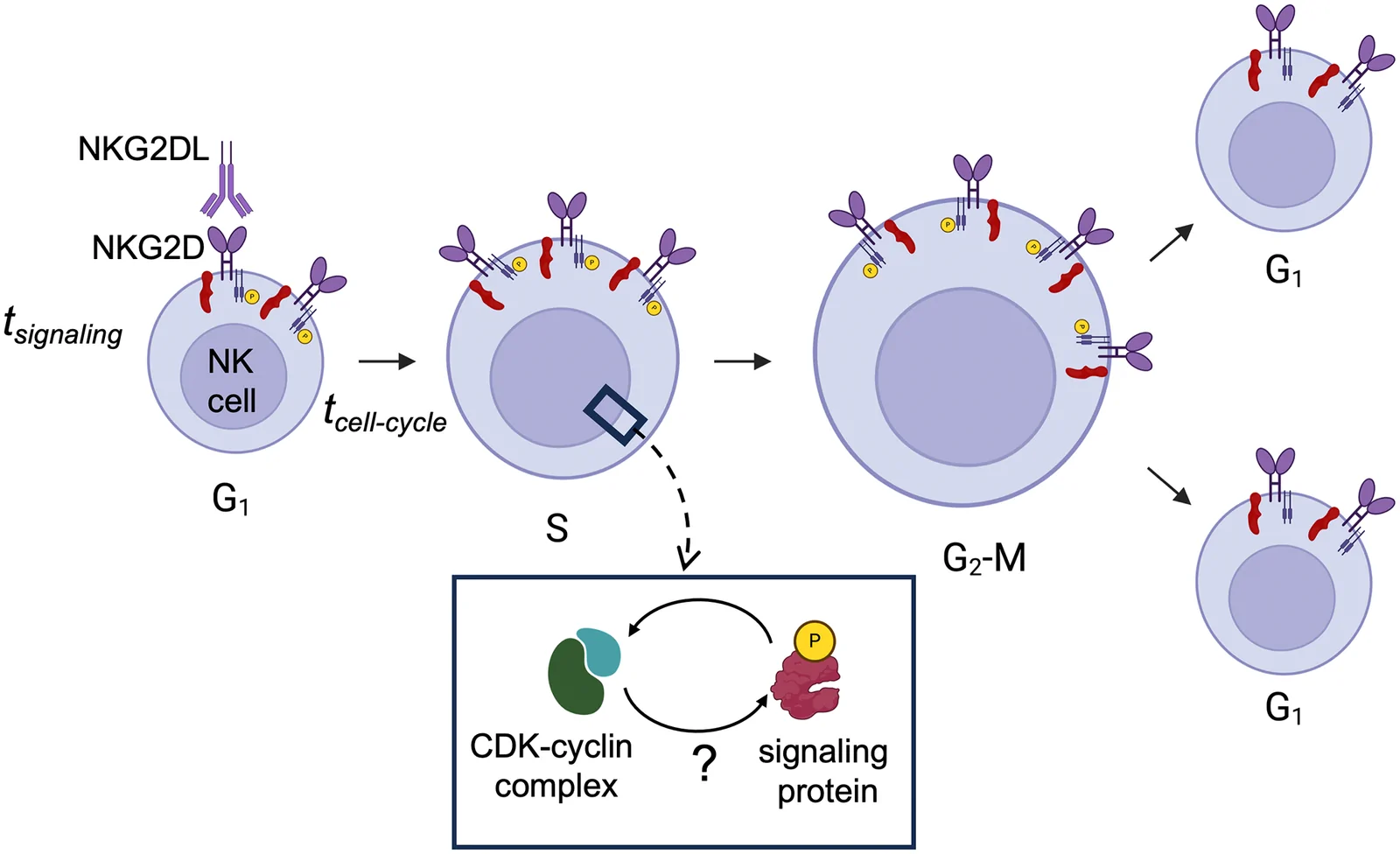
Potential influences of cell cycle transitions on NKG2D receptor signaling kinetics in NK cells. Schematic depiction of potential mechanisms with which cell cycle transitions can regulate NKG2D receptor signaling. As an NK cell progresses through cell cycle stages, the abundances of many proteins including ones involved in NKG2D signaling can increase. The time scale (*t*_*signaling*_) for cell signaling is typically in seconds to minutes and can be 10-100 times smaller than the timescales (*t*_*cell-cycle*_) of cell cycle transitions, which can take hours to occur. The cell volume increases with cell cycle progression as well. The combined effect of increasing protein abundances and cell volume can increase or decrease the frequency at which second-order biochemical signaling reactions occur. This can result in different abundances of signaling products arising from second order signaling reactions in different cell cycle stages. In addition, cell cycle regulating CDK-cyclin complexes can potentially influence post-translational modifications of signaling proteins in specific cell cycle stages affecting NKG2D signaling kinetics. Figure created in BioRender. Das, **J.** (2026) https://BioRender.com/j90h921.

**Fig 2:**
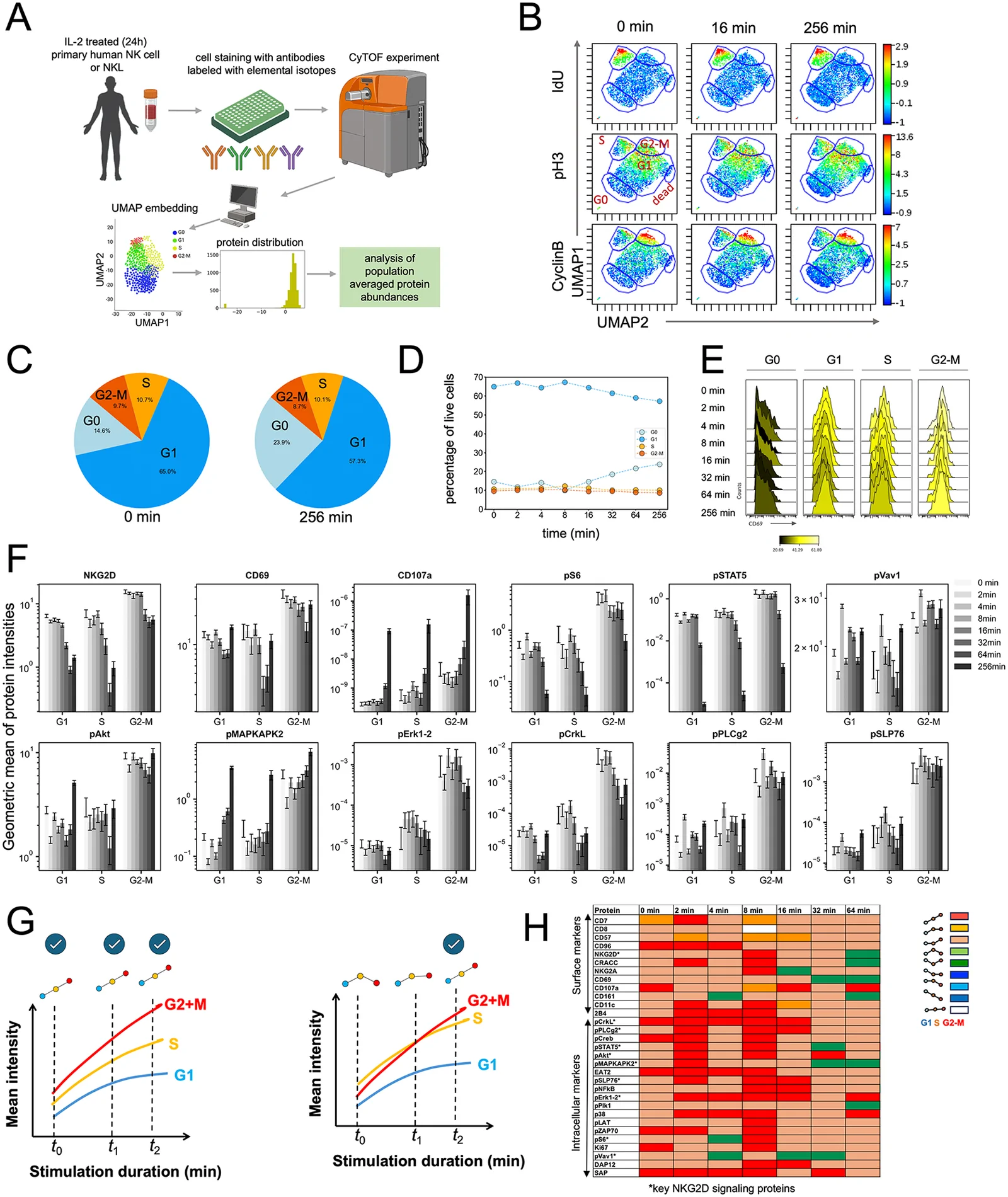
Variation of the abundances of signaling proteins in NKG2D-stimulated primary human NK cells residing in different cell cycle stages. (A) IL-2 treated primary CD56^dim^ NK cells were obtained from a healthy donor and analyzed by CyTOF at 0 (i.e., unstimulated), 2, 4, 8, 16, 32, 64, and 256 min post-NKG2D stimulation. Cell populations were gated using UMAP embeddings into various cell cycle stages for different time points. We log-transformed the protein intensity data to compare mean protein intensities across cell populations at different cell cycle stages (e.g., G1, S, and, G2-M). We used one-sided t-test for the comparison. Figure created in BioRender. Nayak, I. (2026) https://BioRender.com/e7kn53w. (B) Cell populations are gated into G1, S and G2-M stages for different time points using cell cycle markers (see Methods). (C) Proportion of cells (in percentage) at different cell cycle stages are shown at 0 and 256 min post-NKG2D stimulation. (D) Proportion of live CD56dim NK cells in G0, G1, S and G2-M stages are shown at the different time points post-NKG2D stimulation. (E) Shows histograms of CD69 intensities for untransformed (raw) CyTOF data at different time points across cell cycle stages G0, G1, S, and G2-M. The colors of the histograms represent the arithmetic mean. (F) Geometric means of protein intensities for significant NKG2D signaling proteins are shown across post-stimulation time points and cell cycle stages G1, S, and G2-M. Error bars represent the standard error of the geometric mean, estimated using the Delta method [58], assuming log-normal protein intensity distributions. This assumption and the use of the geometric mean as a metric, were evaluated using Kolmogorov-Smirnov test [59] with Bonferroni correction [60] across all 744 protein distributions (31 proteins across 8 time points and cell cycle stages), with ~60% of cases consistent with a log-normal model. (G) Schematic diagram illustrating the comparison of signaling kinetics across cell cycle stages using snapshot data collected at any given time point. (Left panel) The mean protein intensity shows the fastest increase with time in G2-M, a moderate increase in S, and the slowest increase in G1. This kinetic behavior is captured by the snapshot pattens of intensities at times t_1_ and t_2_, i.e., monotonic increase for G1→ S→G2-M. However, the magnitudes in protein intensities in the unstimulated state at t_0_ can influence these relations. For example, in the right panel, the mean protein intensities at different cell cycle stages increase identically over time as those in the left panel but the magnitudes t_0_ differ. Here the snapshot pattens of the intensities at times t_0_ and t_1_ are non-monotomic; highest in the S stage compared to G1 or G2-M, and the snapshot pattern of intensity at t_2_ (monotonic increase) reflect the correct kinetic behavior. Intensities at an earlier time (t_1_), however, do not fully capture this pattern because they are influenced by the intensities across cell cycle stages at t_0_. These trends, derived from CyTOF snapshot measurements, are systematically summarized across multiple post-stimulation time points in Fig H. (H) The variations of the mean log_e_ (Intensity) of surface and intracellular proteins across cell cycle transitions, G1→ S, and S → G2-M, are shown at a fixed time point for different types of variations. A change (increase or decrease) is marked when the change in the mean log_e_ (Intensity) from G1→ S, or S→G2-M, has a statistical significance level α = 0.05 in one-sided t-test. The nine types are characterized by the changes in (G1→ S, S → G2-M) as follows: (increase, increase), (increase, no-change), (no-change, increase), (increase, decrease), (decrease, increase), (decrease, no-change), (no-change, decrease), (decrease, decrease), (no-change, no-change).

Different patterns of variations of protein levels in G1, S, and G2 - M stages at a fixed time point have been used in phosphoproteomic studies [16,17] to characterize cell cycle dependence of protein production and cell signaling. Here we aimed to identify specific patterns of variations of mean protein intensities across cell cycle stages at any time point in the signaling kinetics (Fig 2G). We categorized the variations into different types based on the change, namely, rise or decay, or no-change, in the average intensities between G1 to S, and S to G2 - M for each time point by performing one-sided *t*-tests (Fig 2H). These categories computed from the time snapshot data can help capture relative changes in the kinetics of protein abundances in cell cycle stages. The majority (~ 93%) of the proteins post-stimulation showed a strictly monotonic increase (i.e., increase for G1 → S and increase for S → G2 - M) or a semi-monotonic increase (i.e., non-change for G1 → S and increase for S → G2-M, or vice versa) across cell cycle stages between 2 to 64 mins signaling kinetics. We excluded the t > 64min time points (e.g., t = 256 min) to restrict the analysis of time snapshot data to time points where signaling kinetics occurs at faster time scales than the duration of the cell cycle stages. Proteins involved in cell signaling that showed monotonic or semi-monotonic behavior post-NKG2D stimulation included pCrkL, pPLCγ2, pAkt, pMAPKAPK2, pSLP76, pNFκB, pErk1-2, pPLK1, p38, pLAT, pZAP70, pS6, and pVav1. Interestingly, 10 proteins including pMAPKAPK2, pPLK1, pS6, and pVav1 also showed non-monotonic behavior (~ 7%) in protein intensities at specific time points post-NKG2D (Fig 2H).

To investigate differences in the interplay between NKG2D signaling and cell cycle progression between NK cell lines and primary NK cells, we analyzed NKG2D signaling kinetics in a human NK cell line, NKL, which constitutively expresses the activating human NKG2D receptor. The NKL cells were stimulated with plate-bound anti-NKG2D agonist antibodies. The time course of mean intensities of NKG2D signaling and surface marker proteins in different cell cycle stages showed cell cycle specific changes (S3A Fig) such as at specific time points the mean pVav1 intensities were lower in G2 - M whereas mean pAkt and CD107a intensities were higher in G2 - M compared to G1 and S stages. Compared to the primary CD56^dim^ NK cells, the mean intensities of signaling proteins such as pS6, pErk1-2, and pAkt showed lower variations across cell cycle stages in the NKLs for the same time points (S3A Fig). CD107a and pMAPKAPK2 exhibited a monotonic increase over time within each cell cycle stage in NKL cells. Next, we summarized these trends using CyTOF snapshots at fixed time points post-NKG2D stimulation, which revealed a similar fraction (~8%) of proteins displaying non-monotonic variations following NKG2D stimulation (S3B Fig) across cell cycle stages in NKL cells compared to the primary CD56^dim^ NK cells.

In addition, we engineered the NKL cells to express the activating mouse receptor Ly49H to study another NKR signaling kinetics that uses both the ITAM-bearing adaptor DAP12 and the DAP10 adaptor, in contrast to human NKG2D receptor signaling, which solely uses the DAP10 adaptor [18,19]. Plate-bound anti-Ly49H agonist antibodies were used to stimulate the Ly49H-expressing NKL cells, and found cell cycle stage specific kinetics of the mean intensities for signaling proteins such as pVav1 and pErk1-2; however, the variations of mean intensities across cell cycle stages for pVav1, pS6, pAkt, and pErk1-2, were less than that in the NKG2D-stimulated CD56^dim^ primary NK cells (S4A Fig). A marked difference compared to the NKG2D-stimulated primary CD56^dim^ NK cells was that negligible changes in the intensities of CD69 or CD107a across cell cycle stages were observed for the majority of the time points during the signaling kinetics of the Ly49H-stimulated NKL cells. CyTOF snapshots at fixed time points post-Ly49H stimulation in NKL cells further revealed a higher fraction (~21%) of signaling proteins displaying non-monotonic changes across cell cycle stages in mean protein intensities, compared with NKG2D-stimulated primary CD56^dim^ NK cells (S4B Fig).

In summary, our results show that NKG2D signaling and activation depend on cell cycle stage. In both primary CD56^dim^ NK cells and NKL cells, the time-course and magnitudes of intensities of signaling proteins and functional markers, including CD69 and CD107a, vary across G1, S, and G2 - M phases, with several responses reaching higher levels in G2 - M. However, comparisons at longer stimulation time points (>1 hour) should be interpreted cautiously, as cells may transition between stages during stimulation. Overall, these findings highlight the importance of cell cycle stage in shaping NK cell signaling responses.

### Development of minimal models that couples first-order cell signaling reactions and cell cycle processes

The changes in the mean intensities of proteins in NKG2D-stimulated NK cells with cell cycle stages can arise due to various mechanisms as cells progress through cell cycle stages, such as (a) changes in the average abundances of interacting signaling proteins (i.e., substrate, kinase, or phosphatase), (b) increase in the cell size (volume), and (c) potential interactions of CDKs with cell signaling proteins. To elucidate mechanisms (or hypotheses) regarding how these processes influence the abundances of signaling proteins at different cell cycle stages, we developed a set of minimal models that consider basic components of biochemical signaling reactions coupled with cell cycle processes. Furthermore, because each of these processes is stochastic in nature, which gives rise to randomness in both the duration of cell cycle stages and the production of signaling proteins, we studied stochastic kinetics of our model systems. Here we first considered models that can be solved analytically and exactly, then developed a stochastic simulation method to simulate the model kinetics and validated it by the analytical calculations kinetics.

In our minimal model, an unphosphorylated protein species *A* (e.g., Vav1, Erk) undergoes post-translational modifications such as tyrosine phosphorylation through a first-order reaction. Such protein phosphorylation reactions are initiated by the stimulation of NK cell receptors (e.g., NKG2D) and cytokine receptors (e.g., IL-2R). The phosphorylated form of *A* (denoted as A*) is dephosphorylated by phosphatases in the cell. The protein species *A* can be produced or degraded in individual cells at different stages of the cell cycle. The synthesis, degradation, phosphorylation, and dephosphorylation of protein species *A* are modeled by zero- and first-order reactions occurring at rates *r, δ*, *r*_p_, and *r*_d_, respectively (Fig 3A). We represent the parameter set {*r*, *δ*, *r*_p_, *r*_d_} by a parameter vector ***θ*** for brevity. This model is amenable to exact analytical calculations, which helps in clearly deciphering the interplay between cell signaling and cell cycle processes. The description of phosphorylation/dephosphorylation reactions by first-order reactions is reasonable when the abundances of kinases and phosphatases are in excess compared to the substrate abundance [20]. The details of the rates for the reactions are provided in the legend of Fig 3A.

**Fig 3:**
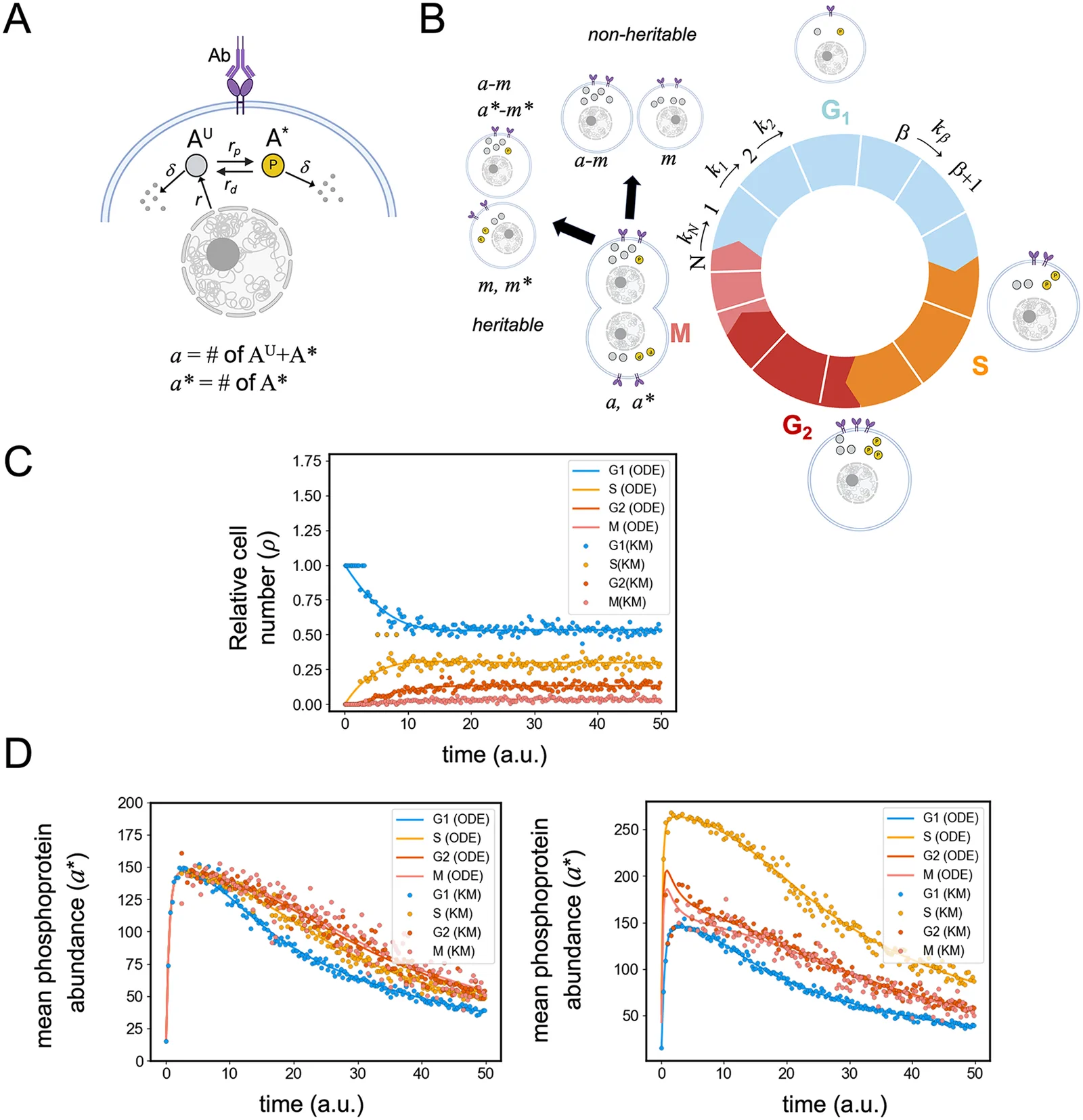
Kinetics of average protein abundances in single cells residing in different cell cycle stages for a first order signaling reaction model. **(A)** Schematic representation of a protein phosphorylation-dephosphorylation model involving first-order reactions. Receptor signaling (implicit) leads to phosphorylation of a protein A^U^ (unphosphorylated form) to A* (phosphorylated form). A^U^ is synthesized inside the cell and both A^U^ and A* can be degraded in the cell. The rates of the reactions are indicated in the schematic. Created in BioRender. Das, J. (2026) https://BioRender.com/kps3mul and https://BioRender.com/wx5stt2. **(B)** Schematic representation of a cell going through different cell cycle stages: G1, S, G2 and M. Each cell cycle stage consists of multiple intermediate stages, where the cell transitions from stage *β* to stage *β* +1 at a rate k_β_. The protein content of the mother cell in the M stage is divided between two daughter cells upon division following a binomial distribution. In the full heritability scenario, phosphorylated proteins in the mother cells are transferred to the daughter cells. In the non-heritability scenario, the phosphorylated forms of proteins are de-phosphorylated upon transfer to daughter cells. Created in BioRender. Das, J. (2026) https://BioRender.com/ong5p0s. **(C)** Shows the population averaged relative cell number or the proportion of the average cell number in each cell cycle stage obtained from Gillespie simulation (circles) or the numerical solution of the ODEs (solid lines). **(D)** Average protein abundances as a function of time in cell cycle stages for the signaling model shown in (**A),** obtained from Gillespie simulation (circles) or the numerical solution of the ODEs (solid lines). The left panel shows the case when the reaction rates are independent of the cell cycle stages, while the right panel shows the case when the phosphorylation rate of A^U^ is increased 10 times in the S stage. We used the following model parameters for the results shown: (left panel) r = r_p_ = r_d_ = 1.0, δ = 0.01, k_β_ = [0.07, 0.1, 0.2, 0.7] in arbitrary units (a.u.), and (right panel) same as in the left panel, except, r_p_=10.0 in the S stage.

We coupled the above signaling reactions, protein production and degradation processes with cell cycle transitions (Fig 3B) in the model. In the model, single cells can be in multiple virtual cell cycle stages {1, 2,..., *β*,…, *N*}, where the cell cycle transitions between these stages are described in the reactions below:


$$\begin{array}{c} \text{stage\textasciitilde{}}\beta \to \text{stage\textasciitilde{}}\beta +1\;\text{(rate\textasciitilde{}}k_{\beta }\mathrm{\backslash textquad}\text{for\textasciitilde{}}1\leq \beta \leq N-1 \end{array}$$ (1a)

$$\begin{array}{c} \text{stage\textasciitilde{}}N\to \text{stage\textasciitilde{}}1+\text{stage\textasciitilde{}}1\;\text{(rate\textasciitilde{}}k_{N}\text{)} \end{array}$$ (1b)

These transitions are described as Markovian [21] processes where the transition to the next stage depends only on the current stage, and the residence time of the single cells in each cell cycle stage *β* follows an exponential distribution. The total number *N* of virtual cell cycle stages and the transition rates {*k*_*β*_} can be chosen to fit experimentally measured probability distribution functions of cell division times [17]. The stages {*β*} describe a collection of intermediate virtual stages that correspond to a specific cell cycle stage (e.g., S stage). The durations of these intermediate virtual stages are exponentially distributed and the durations of cell cycle stages consisting of multiple of these virtual stages follow an Erlang distribution [22]. This construct effectively models the residence times of cell cycle stages observed in experiments [22].

Mathematical models using multiple virtual cell cycle stages have been widely used to describe cell cycle kinetics [23–25]. Several of these models consider cell cycle transition rates proportional to the cell volume with a power (∝ V(t)^q^) [26] to generate size homeostasis where the cell sizes in a dividing cell population maintains an average cell size across generations. The size homeostasis has been observed in experiments with bacterial, yeast, and some mammalian cells [27,28]. These models investigate implications of three major paradigms [27, 28] proposed to explain cell size homeostasis, in which cells divide after increasing their volume at birth by a fixed amount (adder model, q = 1), upon reaching a critical cell size (sizer model, q=∞), or after a specific time interval (timer model, q = 0). Bacterial and yeast cells appear to follow a mixture of the above paradigms (e.g., sizer-like or timer-like) to implement cell size homeostasis [27]. Some of these models also consider mRNA and protein production using reduced gene regulatory models to produce homeostasis of mRNA and protein concentrations (# of mRNA/protein molecules per cell/cell volume) [26,29]. However, concentration homeostasis is usually observed for the total mRNA or protein content [28], and individual mRNA [30] or protein [31] concentrations may show increase (or super scaling), decrease (or sub scaling), or no-change (or scaling) with cell size [31].

Here, we focused on the effect of cell cycle transitions on NK cell signaling occurring in a substantially shorter time duration (0-64 mins, Fig 2) compared to the NK cell division times (~ 30 hrs), thus during cell signaling, a single NK cell is less likely to undergo multiple cell divisions. Therefore, we considered a simplified model where the above cell volume dependent transition rates or protein production rates are not explicitly considered. The effect of the cell cycle stage on protein production/degradation are implemented as cell cycle stage-specific protein production/degradation rates. In our model, as a single cell transitions from the current stage *β* to the next cell cycle stage *β* + 1, the abundances of unphosphorylated and phosphorylated forms of *A* in stage *β*, given by (*a*^U^)_*β*_ and (a^*^)_*β*_, respectively, are fully transitioned to the stage *β* + 1. When the mother cell splits into two daughter cells, described by the *N* → 1 transition, the total number of *A* molecules in a single cell in the terminal cell cycle stage *N* (i.e., *a*_*N*_ *=* (*a*^U^)_*N*_ + (a^*^)_*N*_) is split into the two daughter cells with abundances *m* and *a*_*N*_*-m* in cell cycle stage *β* = 1, with a binomial probability ${(\propto \left(\frac{\text{1}}{\text{2}}\right)}^{m}{\left(\frac{\text{1}}{\text{2}}\right)}^{a_{N}-m})$. This assumption has been supported by experiments and has been incorporated into mathematical models describing cell cycle kinetics [32,33]. Single cell experiments in yeast cells responding to stress have shown the heritability of phosphorylated forms of proteins during mitosis [34]; therefore, we incorporated this possibility in our model. The transition of the phosphorylated protein abundance (a^*^)_*N*_ in stage *N* to the daughter cells is considered in two ways (Fig 3B): (1) No heritability: The newly generated daughter cells at stage 1 do not retain any phosphorylated A* molecule from the mother cell at stage *N*. (2) Full heritability: The number of phosphorylated A* molecules (or a*) in the mother cell at the time of cell division is split into m* and (a*)_*N*_ -m* in the daughter cells, with a binomial probability ${(\propto \left(\frac{\text{1}}{\text{2}}\right)}^{m^{*}}{\left(\frac{\text{1}}{\text{2}}\right)}^{{\left(a^{*}\right)}_{N}-m^{*}})$ (Fig 3B).

The time scales pertaining to protein phosphorylation and dephosphorylation occur on the order of seconds to minutes [4,5,35,36], whereas mammalian cells usually duplicate in 24 hours [37]. In such cases, the time scales for progression in cell cycle stages and signaling kinetics appear to be well-separated. However, the synthesis and degradation of some proteins can occur over longer time scales that can overlap with these ranges; e.g., protein production can take anywhere from ½ hour to tens of hours and protein degradation via ubiquitylation can occur within ~1 hour to a few hours [38]. Further details regarding these time scales are provided in Table 1 and S1 Table. Furthermore, the protein synthesis and degradation rates can vary across cell cycles stages, and CDK proteins can actively participate in post-translational modifications of signaling proteins - all of which could contribute to variations in the abundances of signaling proteins across cell cycle stages. To investigate the roles of these different mechanisms, we considered two hypotheses (*neutral* and *active*) regarding potential mechanisms of cell cycle dependence of synthesis, degradation, phosphorylation, and dephosphorylation rates of signaling proteins in our models. (1) *Active hypothesis*: Here the rates vary ({***θ***^*β*^}) depending on cell cycle stages *β,* representing active participation of cell cycle processes in regulating signaling protein production and signaling kinetics. (2) *Neutral hypothesis*: In this case, the rates {***θ***^*β*^} are constant (=***θ***) across all cell cycle stages, in contrast to the active hypothesis, and represent no dependence of protein synthesis/degradation/signaling on cell cycle stages. In the next section, we use the neutral hypothesis to assess the extent of variation in signaling protein abundances across cell cycle stages arising from changes in the propensity of a second order signaling reaction, which is oppositely affected by increases in protein abundance and cell volume as the cell cycle progresses.

**Table 1:** Estimation of model parameter values.

Parameter	Values	Notes
k_1_, k_2_, k_3_, k_4_	k_1_ ~ 0.07 hr^-1^, k_2_ ~ 0.1 hr^-1^, k_3_ ~ 0.2 hr^-1^, k_4_ ~ 0.7 hr^-1^.	NKL cells grow in the presence of IL-2 in vitro with a doubling time of 24-48 hrs [8]. Primary NK cells obtained from healthy adults expand ex-vivo in the presence of artificial antigen presenting cells and IL-2 with a doubling time of about 30 hours [61]. However, the cell cycle residence times in G1, S, G2, and M are not known. We used the cell cycle residence times of HeLa cells in G1, S, G2, and M stages which are ~ 10 hr, 6 hr, 3 hr, and 1 hr, respectively [7], and the total cell cycle duration of 20 hours to evaluate the fractions of the time spent spent in each cell cycle stage. We assumed NK cells roughly follow the same proportions of cell cycle durations across these stages. If we assume k_1_ to k_4_ correspond to the durations of G1, S, G2, and M stages, then the estimated rates k_1_ to k_4_ for NK cells are for the above case are 0.07 hr^-1^, 0.1 hr^-1^, 0.2 hr^-1^, and 0.7 hr^-1^.
r	10800 - 3240000 molecules hr^-1^	The values are based on measurement of protein turnover in activated CD4 + T cells [62,63]. The synthesis rates show large variations, e.g., CD69 synthesis rates is ~ 180 molecules per min in activated CD4 + T cells whereas β-actin shows a high synthesis rate of 54000 molecules/min.
δ	0.03 - 0.9 hr^-1^	Lysosome and proteasome mediated degradation occur with shorter time scales (half-lives 45min - few hours in HeLa cells) whereas loss of protein abundance due to dilution (or cell replication) occurs in cell cycle time scales (e.g., > 22hr for HeLa cells). Therefore, protein decay rates vary from 0.03-0.9 hr^-1^ in HeLa cells [64]. In activated CD4 + T cells some proteins (e.g., β_2_M) showed fast turn-over with a half-live (t_1/2_) of 45 mins, proteins such as CD3ϵ turned over in t_1/2_ ~ 11 hours, and proteins like histones showed t_1/2_ > 1000 hour turn over times [62,63]. The half-lives for protein turnover reflect degradation time scales [62,63].
r_p_	36-7200000 hr^-1^	The rates are ~ 0.01-2000 s^-1^ [4,5,35,36].
r_d_	36-7200000 hr^-1^	The rates are ~ 0.01-2000 s^-1^ [4,5,35,36]
k_on_	10^5^-10^8^ M^-1^ s^-1^	The association range for protein-protein interaction is 10^5^–10^8^ M^-1^ s^-1^ [65]. In T cell, the association rate of ZAP70 to the ITAM is 5 μM^-1^ s^-1^ = 5 uM^-1^ s^-1^ = 29.90 μm^3^ h^-1^ per molecule [66].
k_off_	450-7200 hr^-1^	The rates are ~ 0.125-2 s^-1^. In T cell, ZAP70 unbinding rate from ITAM 0.125 s^-1^ [66]. SHP-1 unbinding rate from ITAM is 1.7 s^-1^ [67].

We set up a Master equation [21] to describe the stochastic kinetics involving cell signaling and cell cycle transitions as described above. The Master equation was then employed to analytically evaluate the kinetics of population-averaged protein abundances in each cell cycle stage (details in the S1 Text, sections 1-4). In parallel, we developed a stochastic simulation approach to numerically simulate the stochastic trajectories of single-cell abundances of *A* and A* in the dividing cells as they progress through cell cycle stages (Fig 3A). The details regarding the algorithm are provided in the Materials and Methods section. In the model, the total number of cells increases continuously with time due to cell division; however, the population-averaged number of cells in any given cell cycle stage *β* relative to the total number of cells across all cell stages, denoted by *ρ*_*β*_ (see in Materials and Methods), reaches a stationary stage after a sufficiently long period. The time evolution of *ρ*_*β*_ for the stages *β* = 1 to *N* in the model is described by a set of coupled non-linear ordinary differential equations (ODEs) (S1 Text, section 5.1)


$$\begin{array}{c} \frac{d\rho_{1}}{dt}=-k_{1}\rho_{1}+k_{N}\rho_{N}(2-\rho_{1}) \end{array}$$ (2a)

$$\begin{array}{c} \frac{d\rho_{\beta }}{dt}=k_{\beta -1}\rho_{\beta -1}-\rho_{\beta }(k_{\beta }+k_{N}\rho_{N})\;\text{for\textasciitilde{}}2\leq \beta \leq N. \end{array}$$ (2b)

The above non-linear ODEs can be solved numerically. We compared the numerical solutions of the ODEs with the numerical simulations of the stochastic kinetics, which showed excellent agreement (Fig 3C). The steady states of the above ODEs can be analyzed analytically. The steady state of the above kinetics (see Materials and Methods section) shows that the longer the duration of a cell cycle stage, the larger the fraction of cells residing in that stage (S1 Text, section 6). The kinetics of the population-averaged abundance of the total amount of protein *A* = *A*^U^ + A* (${=a}_{\beta }$and phosphoprotein A* (=${\;a}_{\beta }^{*})$ in any given cell cycle stage *β* depends on the rates (***θ***^*β*^) of synthesis, degradation, phosphorylation, and de-phosphorylation in stage *β*, as well as on the cell cycle transition rate from *β* - 1 → *β*, and the fraction of the cells in both the previous stage (*β*-1 for *β* > 1, and, N for *β* = 1) and the current (stage *β*) cell cycle stage. The kinetics of $a_{\beta }$ (S1 Text, section 5.2) is given by,


$$\begin{array}{c} \frac{da_{1}}{dt}=r^{\left(1\right)}-\delta^{\left(1\right)}a_{1}+k_{N}\;\left(\frac{\rho_{N}}{\rho_{1}}\right)(a_{N}-2a_{1}) \end{array}$$ (3a)

$$\begin{array}{c} \frac{da_{\beta }}{dt}=r^{\left(\beta \right)}-\delta^{\left(\beta \right)}a_{\beta }+k_{\beta -1}\;\left(\frac{\rho_{\beta -1}}{\rho_{\beta }}\right)(a_{\beta -1}-a_{\beta })\;\text{for\textasciitilde{}}2\leq \beta \leq N, \end{array}$$ (3b)

and the kinetics of $a_{\beta }^{*}$ (S1 Tex**t**, section 5.3) with full heritability of a* is given by,


$$\begin{array}{c} \frac{da_{1}^{*}}{dt}=r_{p}^{\left(1\right)}a_{1}-\;\left(\lambda^{\left(1\right)}+2k_{N}\frac{\rho_{N}}{\rho_{1}}\right)a_{1}^{*}+k_{N}\frac{\rho_{N}}{\rho_{1}}\;a_{N}^{*} \end{array}$$ (4a)

$$\begin{array}{c} \frac{da_{\beta }^{*}}{dt}=r_{p}^{\left(\beta \right)}a_{\beta }-\;\left(\lambda^{\left(\beta \right)}+k_{\beta -1}\frac{\rho_{\beta -1}}{\rho_{\beta }}\right)a_{\beta }^{*}+k_{\beta -1}\frac{\rho_{\beta -1}}{\rho_{\beta }}\;a_{\beta -1}^{*}\;\text{for\textasciitilde{}}2\leq \beta \leq N. \end{array}$$ (4b)

Where $\lambda^{\left(\beta \right)}=r_{p}^{\left(\beta \right)}+r_{d}^{\left(\beta \right)}+\delta^{\left(\beta \right)}\;$ for 1 ≤ *β* ≤ *N*.

Here, the rates of protein synthesis, degradation, phosphorylation, and dephosphorylation are denoted by $r^{\beta },\;\delta^{\beta },\;r_{p}^{\beta }$ and $r_{d}^{\beta },$ respectively, in stage *β*. The above system of non-linear ODEs can be solved numerically, and the steady-state behavior can be analyzed analytically (see Materials and Methods, S1 Text, section 5). The numerical solution of the above ODEs showed excellent agreement with our stochastic simulations (Fig 3D). Below, we describe the dependencies of the average abundances of *A* and A* in the steady state on the kinetic rates pertaining to the cell cycle, protein synthesis and degradation, and phosphorylation and dephosphorylation processes. The steady state analysis describes long-term stable cellular behavior, whereas NK cell receptor signaling (e.g., NKG2D) is dynamic, and typically occurs in a transient state before steady state is reached. However, NK cells grown in vitro continuously in cytokine-rich [39] culture and with feeder cells [40] may reach a stable state where the NK cells have undergone many divisions and cytokine signaling proteins and behave like a steady-state system. This situation can make steady-state analysis appropriate for the models we describe.

In the absence of any cell cycle transitions, the steady-state values of a and $a^{*}$ are fully determined by the synthesis and degradation rates *r* and *δ*, and phosphorylation and dephosphorylation rates, *r*_*p*_ and *r*_*d*_, *i.e.*, $a=\frac{r}{\delta }\;$ and $a^{*}=\frac{ar_{p}}{r_{p}+r_{d+\;\delta \;}}$ (see Materials and Methods, Eqs. 12a-b and 13a-b). In the presence of cell cycle transitions, the steady state values of a and $a^{*}$ in each cell cycle state in addition on the cell cycle transition rates ${\{k}_{\beta }\}$ (Eqs. 12a-b and 13a-b). The steady state does not depend on the fractions of the cells residing in different cell cycle stages at t = 0. Here we make a few general observations from our steady state analysis. (i) *Neutral hypothesis*: In this case, protein synthesis/degradation and phosphorylation/dephosphorylation rates do not change with cell cycle stages, i.e., $r^{\left(\beta \right)}=r$, $\delta^{\left(\beta \right)}=\delta ,{\;r}_{p}^{\left(\beta \right)}=r_{p},\;$and, ${\;r}_{d}^{\left(\beta \right)}=r_{d}$. When the time scale of protein degradation is smaller than that of the cell cycle transition times, i.e., *δ* < *k*_*β*_, the average abundance per cell $a_{\beta }$ increases monotonically as the cell progresses through the cell cycle stages, and $a_{\beta }^{*}$ follows the increase in $a_{\beta }$ as the cells advance through cell cycle (Fig 3D (left), Eqs. 12a-b and 13a-b, S1 Text, Eqs. 68 and 70). However, when the rate of protein degradation is substantially larger than the cell cycle transition rates, i.e., $\delta \gg k_{\beta }$, the average protein abundances per cell $a_{\beta }\;$are roughly equal in each cell cycle stage, and $a_{\beta }^{*}$ also remains unchanged across *β*. (ii) *Active hypothesis*: When the rates *r*, *δ*, *r*_p_, and *r*_d_ depend on the cell cycle stages, *a*_*β*_ and $a_{\beta }^{*}$ can have non-monotonic variations with progressing cell cycle stages (Fig 3D (right)).

In summary, we developed a stochastic minimal model of signaling kinetics, described by first order biochemical reactions, coupled with cell cycle transitions. We analyzed both the time-dependent and steady-state behavior of the average abundances of the signaling proteins using analytical and numerical approaches. The results from the analysis allowed us to draw a few general trends (monotonic vs. non-monotonic across cell cycle stages) in the variation of the average protein abundances. The analytical calculations also enabled us to validate our algorithm for generating stochastic trajectories by comparing with results (Eqs. 2-4) derived from the Master equation.

### Modeling coupling of non-linear biochemical signaling reactions with cell cycle processes

Biochemical signaling reactions such as phosphorylation by kinases and dephosphorylation by phosphatases are enzymatic in nature and cannot be well approximated by first order reactions when the enzyme concentrations are small compared to that of its substrates [41]. We studied a minimal enzymatic phosphorylation of a substrate here that involved a second order reaction. The model cannot be solved analytically; therefore, we used stochastic simulations of the model for our investigation. We considered phosphorylation of a substrate protein species A (e.g., S6) by a kinase E (e.g., pErk), where the kinase binds to the substrate via a second-order reaction (rate *k*_*on*_) to form an intermediate complex C (EA). The substrate can either dissociate from the complex (rate *k*_*off*_) or become phosphorylated (rate *r*_*p*_) (Fig 4A). The phosphorylated substrate A* is dephosphorylated (rate *r*_*d*_) by phosphatases, which is modeled as a first-order reaction. The above reactions are common in many NK cell receptors including NKG2D receptor-initiated signaling. Because the propensity of a second-order reaction, which determines how frequently the reaction occurs, decreases with increasing cell volume (V) as ∝ 1/V, we incorporated the increase in the volume of a single cell in our stochastic simulation as it progresses through the cell cycle. We also considered for the production (rate *r*) and degradation (rate *δ*) of the substrate A, and the kinase E in the different cell cycle stages, which are modeled by zero- and first-order reactions. The changes in the abundances of A, A*, E, and the intermediate complexes EA as cells transition through cell cycle stages are modeled in the same way as described in the previous section. Further details are provided in the Materials and Methods section.

**Fig 4:**
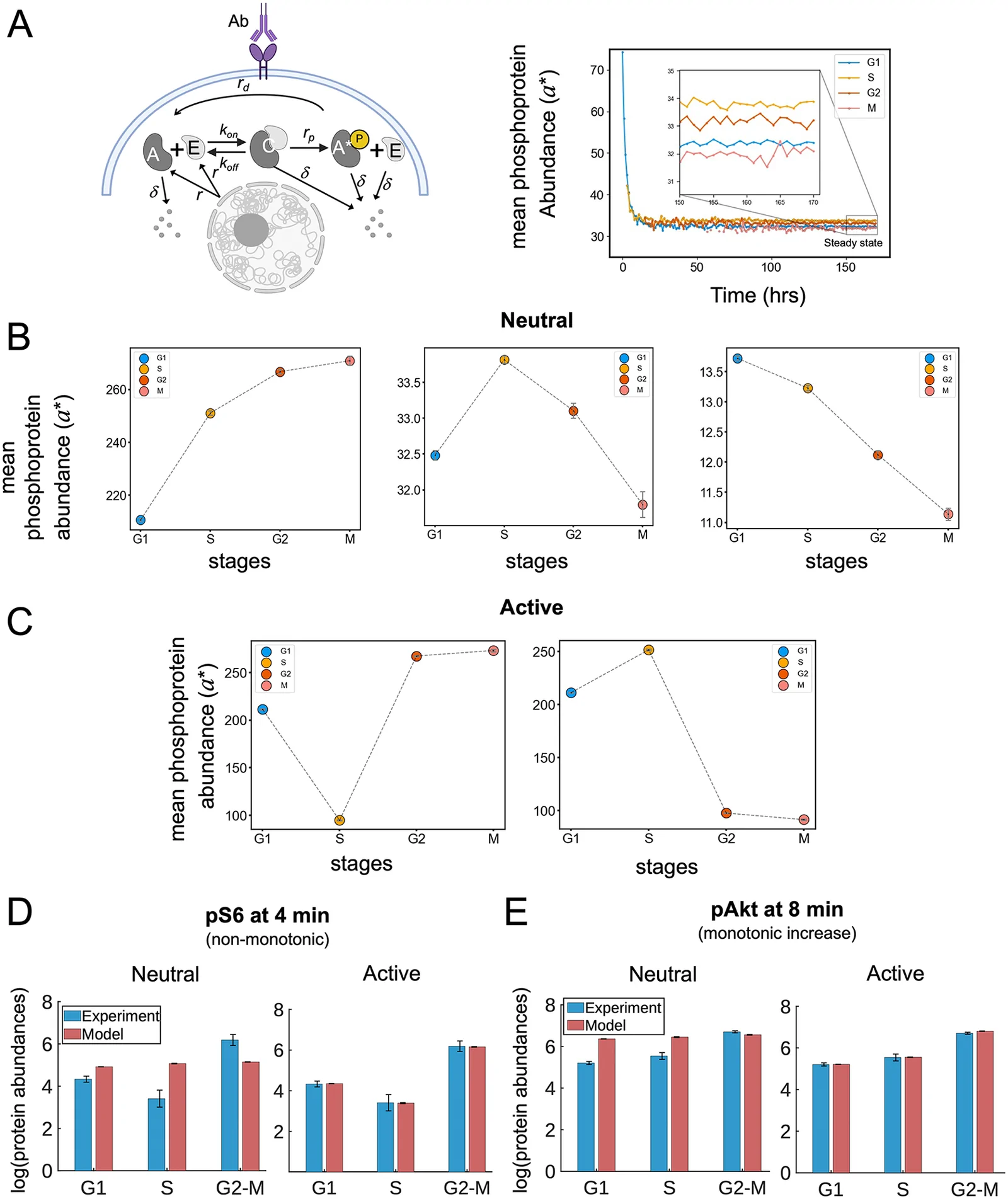
Kinetics of average protein abundances in cells residing in different cell cycle stages for a non-linear signaling reaction model. **(A)** (Left) Schematic representation of a non-linear signaling reaction model. Enzyme E and its substrate A bind to form a complex C, that gives rise to phosphorylated protein A* and the enzyme E is released. The proteins A, E, C, and A* can be degraded, and the proteins A and E are produced in the cell with zeroth-order reactions. Rates associated with the reactions are shown above the arrows. Created in BioRender. Das, **J.** (2026) https://BioRender.com/kps3mul. (right) The mean phosphoprotein abundance per cell (a*) over time [0, 170 hours], across different cell cycle stages, is shown based on simulations of the non-linear model illustrated in (A, left) for a set of model parameters. The inset shows the steady-state protein abundances for each cell cycle stage. **(B-C)** Show the average abundances of A* at the steady state for the signaling model in (A, left) at cell cycle stages G1, S, G2, and M. The results are shown for the *neutral* (**B**) and the *active* **(C)** scenarios. The *neutral* hypothesis can give rise to monotonic increase (B, left), non-monotonic change (B, middle), and monotonic decay (B, right) in the average abundance of A* in the steady state across the cell cycle stages G1 → S → G2→-> M. The non-monotonic change (B, middle) or the monotonic decay (B, right) can be observed in the *neutral* hypothesis within a narrow model parameter range. In the *active* hypothesis*,* the rate of protein phosphorylation is decreased 100-fold in the S stage (C, left) relative to the parameters used in (B, left). This change produces a non-monotonic change in the average abundance of A* (a decrease followed by an increase) for G1 → S → G2. Increasing the rate of phosphorylation of A* 100 times in the S stage and reducing it 100 times in the G2 and M stages relative to the parameters used in (B, left) generates another type of non-monotonic change (an increasefollowed by a decrease) in the average abundance of A* with progressing cell cycle stages for G1 → S → G2 (C, right). All error bars represent the standard error of the mean (SEM). The variations in the mean phosphoprotein abundances between two consecutive cell cycle stages are all statistically significant (*P*-value < 0.05 in *t-*test). The parameter values used and the *P*-values calculated from *t-*tests are provided in S2 Table. **(D)** The mean of logarithmic_10_(abundances) of pS6 at G1, S, and G2-M stages are shown for experiment (blue bar) and model (red bar) at 4 min. (Left) Shows the model fit for the *neutral* hypothesis with a single parameter is used for the fitting (best fit estimate,$\hat{r_{p}}$=456.0 hr^-1^). (Right) Shows the model fit for the *active* hypothesis with four different r_p_ fitting parameters, describing the phosphorylation in different cell cycle stages. The best fit estimates are $\hat{r_{p}}$= [254.0, 87.0, 1306.0, 1306.0] hr^-1^ at cell cycle stages G1, S, G2, and M, respectively. **(E)** The mean of logarithmic_10_(abundances) of pAkt at G1, S, and G2-M stages are shown for experiment (blue bar) and model (red bar) at 8 min. (Left) Shows the model fit for the *neutral* hypothesis with a single parameter $\hat{r_{p}}$= 1464.0 hr^-1^ or the *active* hypothesis (right) with four fitting parameters denoting r_p_ at different cell cycle stages. The best fit estimate is given by $\hat{r_{p}}$= [416.0, 534.0, 1822.0, 1822.0] hr^-1^ at cell cycle stages G1, S, G2, and M, respectively. The error bars in (D-E) represent the standard error of means computed using the of logarithmic of protein abundances. The parameters used in the model are r = 5350 molecules hr^-1^, δ = 0.03 hr^-1^, k_on_=4500 hr^-1^ molecule^-1^, k_off_ = 450.0 hr^-1^, r_d_ = 2500 hr^-1^ and $k_{\beta }$ = [0.07, 0.1, 0.2, 0.7] hr^-1^ (details in S1 Table).

We then simulated the above reactions shown in Fig 4A (left) to evaluate the variation of the cell population-averaged abundances of A* (= $a_{\beta }^{*}$) across cell cycle stages and determine underlying mechanisms. We first performed an analysis of the steady state of the system using our stochastic simulations and investigated mechanisms when the model included the *neutral* or the *active* hypothesis. We observed the following trends in the variations of the per cell average abundances ${\{a}_{\beta }^{*}\}$ in the steady state as cells progress through cell cycle stages (details in S1 Text, section 11 and S3 Table).

- (a) Monotonic increase: For the model with *neutral* hypothesis, the average abundance of A* (= $a_{\beta }^{*}$) increases from G1 → S → G2 - M when the time scales for protein degradation (∝ 1/δ) is similar or larger than that of the cell cycle transition time scales, i.e., 1/*δ* ≥ {1/*k*_*β*_}. In this situation, the total abundances of the substrate, intermediate complex and product (A_total_ = A + A* + EA), and the enzyme (E_total_ = E + EA) increase as the cell progresses through cycle stages, with each cell cycle stage accumulating the abundances from previous stage. Consequently, as the substrate and the enzyme abundances increase, this leads to greater abundances of A* in successive cell cycle stages compared to the previous one (Fig 4B (left)). In contrast, for the model with *active* hypothesis, we can achieve this behavior for a wider range of rate parameters, e.g., when the rates of protein synthesis/phosphorylation increase with increasing cell cycle stages, the kinetics can generate a monotonic increase regardless of the degradation rate.
- (b) Monotonic decrease: This pattern occurs in the model with *neutral* hypothesis when the rate of protein degradation is much faster compared to the cell cycle transitions (*δ* ≫{*k*_*β*_}). In this scenario, the average per cell abundances of A_total_ and E_total_ reach a steady state at each cell cycle stage and thus do not vary across cell cycle stages. However, as the cell volume increases with cell cycle progression, the propensity for the second-order reaction producing the intermediate complex, EA, decreases, leading to the decrease of the abundance of A* (Fig 4B (right)). As the cell volume increases by approximately a factor of two before division, the monotonic decrease in the abundance of A* observed in Fig 4B (right) is ~ 0.23-fold across all cell cycle stages. This point is further discussed in the S1 Text, section 11. For the model with *active* hypothesis, a decrease (or increase) in the synthesis (or degradation) rate or a decrease (or increase) in protein phosphorylation (or dephosphorylation) rate with cell cycle progression can give rise to a substantially large monotonic decrease.
- (c) Non-monotonic changes: This behavior can occur in the model with *neutral* hypothesis for a narrow range of parameter values within those provided in S1 Table. For instance, this situation can arise when the increase in the propensity of the second-order reaction (e.g., protein binding: E + A → EA) yielding the intermediate complex EA due to the increase in the total abundances of E (E_total_) and A(A_total_) with progressing cell cycle stages is counteracted by the decrease in propensity caused by the increasing cell volume (see S1 Text, section 11 for further details). For specific values of r, δ, and the biochemical signaling rates, we can obtain an increasing abundance of A* from G1 → S and then decrease from S → G2 - M (Fig 4B (middle)). However, the magnitudes of protein variation are small ~ 0.06-fold across all cell cycle stages (Fig 4B (middle)). The non-monotonic changes are more easily obtained in the model with *active* hypothesis by cells introducing cell cycle-dependent rates of protein synthesis, or degradation, or biochemical signaling rates (Fig 4C). The sensitivity of the extent of phosphoprotein variation (monotonic vs. non-monotonic) to model parameters is further discussed in S1 Text, Section 9.
- (d) Unchanged abundances: This behavior can be obtained in the model with *neutral* hypothesis when the degradation of E and A are faster compared to the cell cycle transitions (*δ* ≫{*k*_*β*_}) that leads to the constant per cell abundances of A* across cell cycle stages when cell volume does not change across these stages. When the growing cell volume is taken into consideration, a high binding affinity between the E and A (or low dissociation constant *K*_*D*_) reduces the effect of increasing cell volume on the reaction propensity and consequently on the abundances of A*. As a result, the protein abundances remain unchanged across cell cycle stages (S1 Text, section 11, S3 Table). Similarly, in the model with *active* hypothesis, the cell cycle dependent synthesis/degradation/biochemical signaling rates can be tuned to counteract the increases or decreases in abundances of A* between consecutive cell cycle stages to produce constant abundances across cell cycle stages.

As an application of our model, we next evaluated whether the protein signaling variation across cell cycle stages in NK cells as measured in our CyTOF experiments at specific post-NKG2D stimulation timepoints are more preferably described by the *active* or *neutral* hypothesis. We chose two types of signaling kinetic patterns: (1) average pS6 shows a non-monotonic behavior; a decrease for G1 → S followed by an increase for S → G2-M at t = 4 min post-NKG2D stimulation and (2) average pAkt increases monotonically from G1 → S → G2-M at t = 8 min post-NKG2D stimulation. We used a non-linear enzymatic phosphorylation model (Fig 4A) for pS6 and pAkt, where the phosphorylation rate, r_p_, is the same (neutral hypothesis) or can vary (active hypothesis) across cell cycle stages. The model is simulated for a small sub-volume (~ 0.132 μm^3^, S1 Table) within the cell where the reactants can be well mixed. Using the distributions of pS6 and pAkt measured in CyTOF at t = 2 min as initial distributions of the number of protein molecules for the product (A*) within the sub-volume, we evolved the abundances of pS6 and pAkt in individual cells until t = 4 min and t = 8 min, respectively, using the model for the *neutral* and *active* hypotheses (see Materials and Methods). The measured abundances of pS6 at 4 min and pAkt at 8 min in the CyTOF experiments were used to estimate the rate constant(s) (r_p_ in the *neutral* hypothesis, and three cell cycle stage-dependent r_p_ values in the active hypothesis) by minimizing the residual sum of squares (RSS) between the model and the data for the arithmetic mean of the log of the abundances (or the log of the geometric mean). Using Akaike Information Criterion (AIC) [42] (see Methods), an approach for model selection, we found that the *active* hypothesis described the measured data at t = 4 min and 8 min better (ΔAIC = -32 for pS6 and -47 for pAkt) compared to the *neutral* hypothesis for both signaling proteins. Therefore, both for monotonic and non-monotonic changes across cell cycle stages, the cell cycle-dependent phosphorylation of these signaling proteins is more consistent with the data.

In conclusion, we investigated how protein phosphorylation kinetics depend on the cell cycle using stochastic simulations of a nonlinear minimal model. Our steady-state analysis demonstrated that the *active* hypothesis could reproduce a broader range of non-monotonic variations in the mean abundance of the phosphorylated protein, across a wider parameter space compared to the neutral hypothesis. By applying AIC-based model selection to CyTOF measurements of protein abundances, we found that models incorporating cell cycle-dependent phosphorylation rates (*active* hypothesis) are more consistent with the experimental data than those assuming constant rates (*neutral* hypothesis).

## Discussion

NK cells proliferate in response to NKR and cytokine receptor stimulations. As dividing NK cells progress through cell cycle stages from G1 → S → G2 → M, the cell volume increases and cells synthesize many proteins, including those involved in NKR and cytokine signaling. These changes can modulate the propensity of signaling reactions and consequently the abundances of signaling products in receptor stimulated NK cells progressing through cell cycle transitions. In this study, we used CyTOF to investigate the kinetics of signaling proteins in IL-2-treated primary CD56^dim^ NK cells at different cell cycle stages following stimulation with plate-bound NKG2D antibodies. The analysis of the signaling kinetics revealed complex and cell cycle stage-dependent nature of NKG2D signaling. Most signaling proteins exhibit uneven and cell stage specific temporal variations in the CyTOF intensities following NKG2D stimulation. For example, pVav1 displays distinct uneven kinetics in each cell cycle stage, with the most pronounced variations in the S stage and attained larger values of the mean intensities in G2 - M stage. Similarly, the kinetics of the early activation marker CD69 shows cell stage-specific time dependence and reached its highest value in the mean intensity in the G2 - M stage. To summarize trends of variation of mean intensities across cell cycle stages, we also examined mean intensities across cell cycle stages at fixed post-stimulation time points. Most proteins (~ 89%) exhibit monotonic or semi-monotonic increases across cell cycle stages, whereas a smaller fraction (~ 9%) display non-monotonic patterns. The time course of the mean intensities in each cell cycle stage varies qualitatively and quantitatively across biological replicates (different donors), reflecting biological heterogeneity in NK cell signaling across donors and experimental batch effects (S10 Fig).

To investigate differences between primary CD56^dim^ NK cells and NK cell lines, we analyzed NKG2D signaling in the human NK cell line NKL, which constitutively expresses NKG2D. Similar to primary CD56^dim^ NK cells, most signaling proteins display uneven and cell stage specific kinetics. However, their trends differ from those observed in primary cells. For example, the kinetics of the mean intensity of pVav1 in NKL are distinct, with the lowest intensities observed in the G2 - M stage compared to the G1 and S stages, whereas in primary CD56^dim^ NK cells, pVav1 mean intensity attains the highest value at the G2 - M stage. Interestingly, pMAPKAPK2 and the degranulation marker CD107a exhibit monotonic increases with time in protein intensities at each cell cycle stage in NKLs, in contrast these proteins display uneven kinetics in primary CD56^dim^ NK cells. We further examined engineered NKL cells expressing the mouse activating receptor Ly49H, which signals through both DAP12 and DAP10 adaptors, in contrast to NKG2D signaling via DAP10 alone. Upon Ly49H stimulation, most signaling proteins display cell stage specific uneven kinetics, while CD69 and CD107a show negligible changes across cell cycle stages compared to primary CD56^dim^ NK cells and NKG2D stimulated NKLs.

We developed a computational model the describes the stochastic kinetics of cell signaling in single dividing cells. The signaling reactions minimally describe phosphorylation/dephosphorylation events in the context of NK cell signaling. The model represents transitions between successive cell cycle stages as a series of fixed-rate steps through multiple intermediate virtual stages. These simplifying approximations make the model amenable to analytical calculations and less intensive computer simulations without sacrificing the generality for analyzing the dependence of signaling kinetics on cell cycle stages. Previous computational models described cell cycle transitions following this strategy and several models in addition considered cell cycle transition rates proportional to the cell volume with a power law dependence [26] to generate size homeostasis where the cell sizes in a dividing cell population maintains an average cell size across generations. Though size homeostasis in dividing cells has been observed in some mammalian cells it is unknown if it holds for NK cells. Our model is analytically tractable when signaling reactions are described by first order reactions, and the steady state analysis uncovered different parameter regimes where the mean abundances of the phosphorylated protein in each cell cycle stage can vary monotonically or non-monotonically with progressing cell cycle stages. When the signaling reactions included second order enzyme-substrate reactions, we analyzed the kinetics numerically through a stochastic simulation algorithm that allowed us to sample probability distributions described by the Master equation for the system. We found that cell cycle transitions give rise to broader steady-state distributions of total protein abundance than predicted by a Poisson distribution (S11 Fig). The simulated stochastic trajectories allowed us to compare different hypotheses for cell cycle regulation of signaling kinetics.

We considered two broad hypotheses regarding the mechanisms of regulation of cell signaling kinetics by cell cycle processes in our models, namely, the *neutral* hypothesis where the protein production, degradation, and signaling biochemical rates are fixed and do not change with cell cycle transitions, and the *active* hypothesis where these rates can vary in different cell cycle stages. Biosynthesis of proteins have been found to be affected by the ribosome content and the metabolic stage of the cell that varies across cell cycle stages [43], thus the *active* hypothesis represents a more realistic case. Our investigations showed the *neutral* hypothesis is only able to qualitatively recapitulate the different types of monotonic and non-monotonic variations of signaling protein abundances with cell cycle stages; however, those occur in a restricted range of model parameters. Evaluation of the *neutral* and the *active* scenarios in describing the CyTOF measured signaling protein abundances for two signaling proteins pS6 and pAkt favors the active hypothesis based on AIC-based model selection. This suggests that even monotonic increases, which might intuitively be explained by the *neutral* hypothesis, are more quantitively consistent with the *active* hypothesis given different rates of changes of phospho-protein abundances across cell-cycle stages. Thus, our model suggests that signaling protein abundances in NKG2D-stimulated dividing NK cells are more accurately described by an active hypothesis through cell cycle-dependent signaling rates rather than by a neutral mechanism.

The time scale NK cell proliferation is about an order of magnitude slower than that (~ 1/mins to 1/secs) of the signaling kinetics - thus the signaling kinetics in a cell cycle stage was mainly influenced by the protein synthesis and degradation rates in the cell cycle stage. However, for lymphocytes such as T cells, the cell division can occur in much shorter (~ 2-4 hours duplication time [44] time scale in the expansion phase. In such cases the cell cycle transition and signaling kinetic time scales can be less separated in magnitude, and the protein synthesis/degradation rates as well as the abundances of phosphorylated forms of the proteins in several preceding stages can influence the signaling kinetics in the current stage. This will be an interesting future direction. We analyzed simple enzymatic signaling reactions in our model. However, certain signaling reactions can involve positive feedback where the product promotes its own production and displays digital response to stimulations, e.g., Ras activation in T- and B- cells showing a digital response depending on abundances of RasGEFs such as SOS1 and Rasgrp1 [45]. As the abundances of substrates and enzymes can vary across cell cycle stages, it would of interest to investigate if such digital response occurs only in specific cell cycle stages [45].

### The limitation of the study

For primary NK cells, expressions of some of the cell cycle marker proteins (e.g., pH3) were low, which made it difficult for us to separate G2 and M stages in gating the cell populations using UMAP embeddings. We manually gated cell populations into different cell cycle stages on the UMAP plane, which resulted in the loss of a small fraction of that cell population at the borders between two cell cycle gates. In this study, we used geometric means to characterize protein intensity variation patterns while minimizing the influence of outliers in protein intensity distributions. Indeed, other metrics that are more sensitive to outliers (e.g., arithmetic mean) can yield different patterns. Because the geometric means for CyTOF measurements that include negative intensities is ill-defined, we assigned a small positive constant to negative values prior to analysis. We found that for some proteins and timepoints, the identification of monotonic (or non-monotonic) changes across cell cycles showed dependence on the choice of the parameters used in CyTOF data analysis, both for geometric and shifted geometric mean (see Methods and S7 Fig). Overall, these tests show that the variation of protein intensities across cell cycle stages can be sensitive to donor-donor variations, and the metric (e.g., geometric mean) used in quantifying the population mean. Thus, to further quantify and validate the cell cycle dependence of signaling proteins further experimentation is needed, for example, measuring CDK abundances upon NK cell stimulation and assessing their impact on biochemical signaling rates along cell cycle stages across donors would be an interesting direction for future studies. The approaches presented here serve as hypothesis generation tools for further experimentation.

We considered four cell cycle stages in simulating the non-linear signaling kinetics to keep the computation time feasible. This makes the duration of the cell cycle stages in the model distributed exponentially; however, the durations of cell cycle stages have been observed to follow an Erlang distribution with substantially less variations than the exponential distribution. Since the cell cycle durations are almost an order of magnitude longer than the cell signaling time scales, we reasoned the variations in the durations caused by the exponential distributions will not affect the qualitative conclusions drawn from the model. Cell cycle-dependent gene regulation [26,46–49], similar to previous modeling efforts, is not considered explicitly, as it is not known if the protein production in NK cells for the measured signaling proteins will follow the generic and simple description of the well-studied gene regulation models. This could be an exciting future direction as more detailed datasets [50] for simultaneous expressions of gene, proteins, and phosphorylated forms of proteins are being generated.

## Materials and methods

### Ethics statement

Human PBMCs were obtained from blood that was procured from the Blood Centers of the Pacific under an Institutional Review Board-approved protocol (IRB #10–00265) at University of California at San Francisco, USA. The donors were not identified in the blood samples obtained from the blood centers.

### Cell preparation and NKR stimulation

Human peripheral blood NK cells were isolated from blood obtained from healthy adult donors, cultured in IL-2 for 24 hours, and then stimulated with plate-bound agonist anti-human NKG2D mAb as described previously [51]. The IL-2-treated NK cells were stimulated with anti-human NKG2D mAb (5 µg/ml, clone 1D11, BioLegend) for 2, 4, 8, 16, 32, 64, and 256 min. Human peripheral blood NK cells were isolated from blood obtained from healthy adult donors, cultured in IL-2 for 24 hours, and then stimulated with plate-bound agonist anti-human NKG2D mAb as described previously. The IL-2-treated NK cells were stimulated with anti-human NKG2D mAb (5 µg/ml, clone 1D11, BioLegend) for 2, 4, 8, 16, 32, 64, and 256 min. The NKL cells used in this study were generated and maintained as described previously [52]. The human NKL cell line, which endogenously expresses NKG2D, was transduced with pMX-puro retroviral vectors containing the mouse Ly49H and positive NKL cells were isolated by using flow cytometry sorting [8]. The NKL cells were stimulated with plate-bound agonist anti-Ly49H (5 µg/ml, clone 3D10, eBioscience) mAbs) or anti-NKG2D (5 µg/ml, clone 1D11, BioLegend) mAbs as described previously [52] for 8, 32, 64, 128, and 256 min. The NKL cells were stimulated with plate-bound agonist anti-Ly49H (5 µg/ml, clone 3D10, eBioscience) mAbs) or anti-NKG2D (5 µg/ml, clone 1D11, BioLegend) mAbs as described previously [52] for 8, 32, 64, 128, and 256 min.

### Mass cytometry

Cell staining and mass cytometry (i.e., CyTOF) were performed as described previously [51]. The mass cytometry Ab panels used for the analysis of primary human NK cells and NKL cells are shown in S4 and S5 Tables, respectively. Cell staining and mass cytometry (i.e., CyTOF) were performed as described previously [51]. The mass cytometry Ab panels used for the analysis of primary human NK cells and NKL cells are shown in S4 and S5 Tables, respectively.

### Gating strategy

Gating of live CD56^dim^ NK cells (i.e., CD45^+^ cisplatin^-^ CD3^-^ CD235^-^ CD61^-^ CD15^-^CD56^dim^ CD16^+^) and live NKL cells (i.e., CD45^+^ cPARP^-^ cisplatin^-^) was done using Cytobank software. Gating of CD56^dim^ NK cell and NKL populations residing in different cell cycle stages was performed using UMAP (nearest neighbor = 15, min distance = 0.01) based on the cell cycle markers Idu, pRb, Cyclin B, pH3, and Ki67, and cell viability marker cisplatin in Cytobank software. We first eliminated dead cells by removing the cisplatin^+^pRb^-^Idu^-^pH3^-^ population. The cell population in the G0 stage was gated based on pRb^-^ Idu^-^ CyclinB1^low^ pH3^-^ expression (S1 Fig). The cell populations in the G1, S, and G2 stages were gated based on pRb^+^ Idu^-^ Cyclin B1^low^ pH3^-^ expression, Idu^+^ pH3^-^ expression, and Idu^-^ Cyclin B1^high^ pH3^-^ expression, respectively (S1 Fig). In NKG2D-stimulated primary NK cells, we did not see a clear distinction of the pH3^+^ expression; therefore, we could not separate the cell population in the M stage from the G2 stage and designated as G2-M stage. (S1 Fig). Whereas in NKL cells, we identified distinct populations of M-stage cells that are pH3^+^pS6^+^pMAPKAPK2^+^ [53], although their percentage was very small. Whereas in NKL cells, we identified distinct populations of M-stage cells that were pH3^+^pS6^+^pMAPKAPK2^+^ [53], although their percentage was very small. Here, we combined these two populations G2 and M into a single G2 + M stage and similarly designated it as the G2-M stage.

### Data processing

We exported the untransformed mass cytometry (.fcs) data after splitting it into cell cycle stages. Our gating yielded cell numbers that varied between ~ 400–900 for the G0, S, and G2-M stages and ~ 3000-4000 for the G1 stage. Next, we examined the distribution of intensities corresponding to the expression of signaling proteins in cell populations residing in different cell cycle stages at various time points. The distributions of intensities are asymmetric and contained expressions at negative values. To compare the geometric mean of protein expression (Fig 2F, S2 Fig) over time across cell cycle stages, or to compute the variation of protein expression across cell cycle stages at a fixed time (Fig 2H, S3 and S4 Figs), we transformed the intensities to a natural log scale (log_e_). We assigned a small constant value ϵ (10^-11^) which is less than the minimum positive intensity (~10^-5^), to zero and negative values, since the logarithm of these values cannot be computed (S5 Fig). We checked sensitivity of the variation of the mean intensities with cell cycle stages to the changes of ϵ and the significance level α used in the *t*-test for primary NK cells vs. NKL cells. The qualitative nature of the variations of protein intensities in Fig 2H did not change for ϵ < 10^-11^ but changed at certain time points for those proteins when ϵ was chosen to be higher (~10^-9^- 10^-6^) (see S7 Fig for details). In addition, decreasing to α < 0.05 led to qualitative changes in intensity variations for some proteins compared to that shown in Fig 2H.

### Statistical tests

From CyTOF snapshots, to determine whether the log-transformed intensities of a signaling protein increased, decreased, or did not change between cell cycle stages (Fig 2H, S3 and S4 Figs), we sorted single cells into cell cycle stages. We then performed a one-sided *t-*test (number of cells > 369 in measured data in Fig 2H) comparing the log-transformed intensities of single cells between two cell cycle stages (e.g., G1 and S) to assess whether an increase occurred between the cell cycle stages (H_1_: μ_2_ > μ_1_). We additionally performed a one-sided *t-*test to determine whether the protein intensities decreased between the cell cycle stages (H_1_: μ_2_ < μ_1_). If neither of these tests achieved the significance level α = 0.05, we classified the protein as having “no change” between the stages. For our stochastic simulation results presented in Fig 4, we used similar *t-*tests to compare the abundances of phosphoproteins at a steady state between cell cycle stages.

### Testing protein variation patterns using a non-parametric test

To verify the robustness of the results presented in Fig 2H, which were obtained using a parametric *t*-test, we further examined the variations of protein intensity patterns across cell cycle stages in CD56^dim^ NKG2D-stimulated primary NK cells using a non-parametric approach based on shifted geometric mean. For this method, the null hypothesis is that the true means of the two intensity distributions are equal (H_0_: μ_1_ = μ_2_), where μ_1_, μ_2_ represent the means of the two distributions. To estimate the *p*-values, we bootstrapped both the observed distributions n_b_ times to calculate the difference between bootstrapped means (Δ = μ_1_^*^- μ_2_^*^), where μ_1_^*^, μ_2_^*^ denote the means computed from protein intensities $x_{i}^{*}$ in bootstrapped samples 1 and 2, respectively. Finally we calculated p values from the distribution of Δ using the formula [54], p-value = 2 ×min(p,1−p), where p = number of (Δ > 0) / n_b_.

Shifted geometric mean: To calculate the shifted geometric mean in bootstrapped samples (S12 Fig), we used $\mu^{*}=\text{exp}\left(\frac{\text{1}}{n}\sum_{i}\text{log}(x_{i}^{*}+c)\right)-c$, where c is a small positive constant, which was set to 10^-11^. This calculation was applied to both μ_1_^*^ and μ_2_^*^.

### Steady state analysis of kinetic equations for the averages of cell populations and protein abundances

We evaluated the kinetics of the total numbers of cells ($n_{\beta }$) in any cell cycle stage β, as well as the total numbers of A (or $A_{\beta }$) and A* (or $A_{\beta }^{*}$) in the cell population residing in the stage β, using the Master equation [55] describing the stochastic kinetics for the transitions shown in Eqns. 1a-b in the main text and Eqns 2-9 in the S1 Text. The details of the derivation are provided in the S1 Text (sections 1–4). Here, we present the ODEs describing the above cell population averaged kinetics which show the couplings between the processes regulating the cell cycle and phosphorylation/dephosphorylation kinetics.

The kinetics for {n_β_} are described by,


$$\begin{array}{c} \frac{dn_{1}}{dt}=-k_{1}n_{1}+2k_{N}n_{N} \end{array}$$ (5a)

$$\begin{array}{c} \frac{dn_{\beta }}{dt}=k_{\beta -1}n_{\beta -1}-k_{\beta }n_{\beta }\;\text{when\textasciitilde{}}2\leq \beta \leq N \end{array}$$ (5b)

The kinetics for A_β_ are given by,


$$\begin{array}{c} \frac{dA_{1}}{dt}=r^{\left(1\right)}n_{1}-\delta^{\left(1\right)}A_{1}-k_{1}A_{1}+k_{N}A_{N} \end{array}$$ (6a)

$$\begin{array}{c} \frac{dA_{\beta }}{dt}=r^{\left(\beta \right)}n_{\beta }-\delta^{\left(\beta \right)}A_{\beta }+k_{\beta -1}A_{\beta -1}-k_{\beta }A_{\beta }\;\text{for\textasciitilde{}}2\leq \beta \leq N \end{array}$$ (6b)

The kinetics for $A_{\beta }^{*}$ when there is *no heritibility* of A* during cell division are given by,


$$\begin{array}{c} \frac{dA_{1}^{*}}{dt}=r_{p}^{\left(1\right)}A_{1}-\;\left(r_{p}^{\left(1\right)}+r_{d}^{\left(1\right)}+\delta^{\left(1\right)}+k_{1}\right)A_{1}^{*} \end{array}$$ (7a)

$$\begin{array}{c} \frac{dA_{\beta }^{*}}{dt}=r_{p}^{\left(\beta \right)}A_{\beta }-\;\left(r_{p}^{\left(\beta \right)}+r_{d}^{\left(\beta \right)}+\delta^{\left(\beta \right)}+k_{\beta }\right)A_{\beta }^{*}+k_{\beta -1}A_{\beta -1}^{*}\;\text{for\textasciitilde{}}2\leq \beta \leq N \end{array}$$ (7b)

For the case of *full heritibility*, the kinetics of $A_{\beta }^{*}$ for β ≥ 2 is same as that above, and for β = 1 it is changed to,


$$\begin{array}{c} \frac{dA_{1}^{*}}{dt}=r_{p}^{\left(1\right)}A_{1}-\;\left(r_{p}^{\left(1\right)}+r_{d}^{\left(1\right)}+\delta^{\left(1\right)}+k_{1}\right)A_{1}^{*}+k_{N}A_{N}^{*} \end{array}$$ (8)

In CyTOF experiments, single cells can be sorted based on expression levels of specific proteins to determine the cell cycle stage that single cell is residing in. Abundances of different protein species can be then evaluated in these sorted cells. However, cytometry experiments do not track changes in the total number of cells that can occur due to cell division. Therefore, the relative numbers of cells in different cell cycle stages, i.e., $\rho_{\beta }=n_{\beta }/(\sum_{\beta }n_{\beta }),\;$ and the average abundance of protein species A in a cell cycle stage β, given by $a_{\beta }=A_{\beta }/n_{\beta }$, can be evaluated from the CyTOF data. The number of cells in each cell cycle stage, {n_β_}, increase exponentially with time, therefore, the total number of proteins in the cell population, {$A_{\beta }$} and, $\left\{A_{\beta }^{*}\right\}$ increase exponentially with time as well. However, the relative numbers of cells in any stage β, given by $\rho_{\beta }=\frac{n_{\beta }}{\sum_{\beta =\text{0}}^{N}n_{\beta }}$ approaches a steady state. The kinetics of {ρ_β_} is given by (see S1 Text, section 5.1 for details),


$$\begin{array}{c} \frac{d\rho_{1}}{dt}=-k_{1}\rho_{1}+k_{N}\rho_{N}(2-\rho_{1}) \end{array}$$

$$\begin{array}{c} \frac{d\rho_{\beta }}{dt}=k_{\beta -1}\rho_{\beta -1}-\rho_{\beta }(k_{\beta }+k_{N}\rho_{N})\;\text{for\textasciitilde{}}2\leq \beta \leq N \end{array}$$ (8b)

The steady state values are given by,


$$\begin{array}{c} \rho_{1}=\frac{2k_{N}\rho_{N}}{k_{1}+k_{N}\rho_{N}} \end{array}$$ (9a)

$$\begin{array}{c} \rho_{\beta }=2\;\left[\prod_{i=1}^{\beta -1}\frac{k_{i}}{k_{i}+k_{N}\rho_{N}}\right]\frac{k_{N}\rho_{N}}{k_{\beta }+k_{N}\rho_{N}}\;\text{for\textasciitilde{}}2\leq \beta \leq N \end{array}$$ (9b)

The average abundances of A and A* in a single cell {*a*_β_} and {a*_β_}, respectively, approach steady states as well. The kinetics of these variables are given by (see **S1 Text**, section 5.2 for details),


$$\begin{array}{c} \frac{da_{1}}{dt}=r^{\left(1\right)}-\delta^{\left(1\right)}a_{1}+k_{N}\;\left(\frac{\rho_{N}}{\rho_{1}}\right)(a_{N}-2a_{1}) \end{array}$$ (10a)

$$\begin{array}{c} \frac{da_{\beta }}{dt}=r^{\left(\beta \right)}-\delta^{\left(\beta \right)}a_{\beta }+k_{\beta -1}\;\left(\frac{\rho_{\beta -1}}{\rho_{\beta }}\right)(a_{\beta -1}-a_{\beta }quad\text{for\textasciitilde{}}2\leq \beta \leq N \end{array}$$ (10b)

For no heritability the kinetics of $\left\{a_{\beta }^{*}\right\}$ are (see **S1 Text**, section 5.3 for details) given by,


$$\begin{array}{c} \frac{da_{\text{1}}^{*}}{dt}=r_{p}^{\left(\text{1}\right)}a_{\text{1}}-\left(\lambda^{\left(\text{1}\right)}+\text{2}k_{N}\left(\frac{\rho_{N}}{\rho_{\text{1}}}\right)\right)a_{\text{1}}^{*} \end{array}$$

$$\begin{array}{c} \frac{da_{\beta }^{*}}{dt}=r_{p}^{\left(\beta \right)}a_{\beta }-\;\left(\lambda^{\left(\beta \right)}+k_{\beta -1}\frac{\rho_{\beta -1}}{\rho_{\beta }}\right)a_{\beta }^{*}+k_{\beta -1}\frac{\rho_{\beta -1}}{\rho_{\beta }}\;a_{\beta -1}^{*}\;\text{for\textasciitilde{}}2\leq \beta \leq N \end{array}$$

For full heritability the kinetics of $\left\{a_{\beta }^{*}\right\}$ are,


$$\begin{array}{c} \frac{da_{1}^{*}}{dt}=r_{p}^{\left(1\right)}a_{1}-\;\left(\lambda^{\left(1\right)}+2k_{N}\frac{\rho_{N}}{\rho_{1}}\right)a_{1}^{*}+k_{N}\frac{\rho_{N}}{\rho_{1}}\;a_{N}^{*} \end{array}$$ (11a)

$$\begin{array}{c} \frac{da_{\beta }^{*}}{dt}=r_{p}^{\left(\beta \right)}a_{\beta }-\;\left(\lambda^{\left(\beta \right)}+k_{\beta -1}\frac{\rho_{\beta -1}}{\rho_{\beta }}\right)a_{\beta }^{*}+k_{\beta -1}\frac{\rho_{\beta -1}}{\rho_{\beta }}\;a_{\beta -1}^{*}\;\text{for\textasciitilde{}}2\leq \beta \leq N \end{array}$$ (11b)

Here, $\lambda^{\left(\beta \right)}=r_{p}^{\left(\beta \right)}+r_{d}^{\left(\beta \right)}+\delta^{\left(\beta \right)}\;$ for 1 ≤ β ≤ N.

The steady state values of {*a*_β_} as functions of the scaled {${\tilde{r}}^{\left(\beta \right)}$} and dimensionless {${\tilde{k}}_{\left(\beta \right)}$} (i.e., ≤ 1) variables are given by (see S1 Text, section 5.2 for details),


$$\begin{array}{c} a_{1}={\tilde{r}}^{\left(1\right)}+{\tilde{k}}_{N}a_{N} \end{array}$$ (12a)

$$\begin{array}{c} a_{\beta }={\tilde{r}}^{\left(\beta \right)}+\sum_{i=1}^{\beta -1}\;\left(\prod_{j=1}^{i}{\tilde{k}}_{\beta -j}\right){\tilde{r}}^{\left(\beta -i\right)}+\;\left(\prod_{j=1}^{\beta -1}{\tilde{k}}_{\beta -j}\right){\tilde{k}}_{N}a_{N}={\tilde{r}}^{\left(\beta \right)}+\sum_{\beta^{\prime }=1}^{\beta -1}R_{\beta \leftarrow \beta^{\prime }}+M_{\beta }a_{N}\;\text{for\textasciitilde{}}2\leq \beta \leq N \end{array}$$ (12b)

and,


$$\begin{array}{c} a_{N}=\frac{\tilde{r}\left(N\right)+\sum_{i=1}^{N-1}\;\left(\prod_{j=1}^{i}{\tilde{k}}_{N\;-j}\right)\tilde{r}\left(N\;-i\right)}{1-\left(\prod_{j=1}^{N-1}{\tilde{k}}_{N-j}\right){\tilde{k}}_{N}} \end{array}$$ (12c)

where,

${\tilde{r}}^{\left(1\right)}=\frac{r^{\left(1\right)}}{\delta^{\left(1\right)}+2k_{N}(\rho_{N}/\rho_{1})}$, ${\tilde{r}}^{\left(\beta \right)}=\frac{r^{\left(\beta \right)}}{\delta^{\left(\beta \right)}+k_{\beta -1}(\rho_{\beta -1}/\rho_{\beta })}$ for 2 ≤ β ≤ N, ${\tilde{k}}_{\beta }=\frac{k_{\beta }\left(\frac{\rho_{\beta }}{\rho_{\beta +\text{1}}}\right)}{\delta^{\left(\beta +\text{1}\right)}+k_{\beta }\left(\frac{\rho_{\beta }}{\rho_{\beta +\text{1}}}\right)}$ for 1 ≤ β ≤ N-1, ${\tilde{k}}_{N}=\frac{k_{N}(\rho_{N}/\rho_{1})}{\delta^{\left(1\right)}+2k_{N}(\rho_{N}/\rho_{1})}$, $R_{\beta \leftarrow \beta^{\prime }}=\left(\prod_{j=\beta^{\prime }}^{\beta -1}{\tilde{k}}_{j}\right){\tilde{r}}^{\left(\beta^{\prime }\right)}$ and $M_{\beta }={\tilde{k}}_{N}\left(\prod_{j=\text{1}}^{\beta -\text{1}}{\tilde{k}}_{\beta -j}\right)$ for 2 ≤ β ≤ N.

The steady state values for {$a_{\beta }^{*}=A_{\beta }^{*}/n_{\beta }$} functions of dimensionless {${\tilde{r}}_{p}^{\left(\beta \right)}$} and {${\tilde{k}}_{\beta }^{\left(p\right)}$} variables are given by (see **S1 Text**, section 5.3 for details),


$$\begin{array}{c} a_{1}^{*}={\tilde{r}}_{p}^{\left(1\right)}a_{1}+{\tilde{k}}_{N}^{\left(p\right)}a_{N}^{*}\text{\textasciitilde{}(full\textasciitilde{}heritability)} \end{array}$$ (13a)

$$\begin{array}{c} a_{\beta }^{*}={\tilde{r}}_{p}^{\left(\beta \right)}a_{\beta }+\sum_{i=1}^{\beta -1}\;\left(\prod_{j=1}^{i}{\tilde{k}}_{\beta -j}^{\left(p\right)}\right){\tilde{r}}_{p}^{\left(\beta -i\right)}a_{\beta -i}+\;\left(\prod_{j=1}^{\beta -1}{\tilde{k}}_{\beta -j}^{\left(p\right)}\right){\tilde{k}}_{N}^{\left(p\right)}a_{N}^{*}={\tilde{r}}_{p}^{\left(\beta \right)}a_{\beta }+\sum_{\beta^{\prime }=1}^{\beta -1}R_{\beta \leftarrow \beta^{\prime }}^{\left(p\right)}a_{\beta^{\prime }}+M_{\beta }^{\left(p\right)}a_{N}^{*}\;\text{for\textasciitilde{}}2\leq \beta \leq N\text{\textasciitilde{}(full\textasciitilde{}heritability)} \end{array}$$ (13b)

for 2 ≤ β ≤ N,

where,

${\tilde{r}}_{p}^{\left(1\right)}=\frac{r_{p}^{\left(1\right)}}{r_{p}^{\left(1\right)}+r_{d}^{\left(1\right)}+\delta^{\left(1\right)}+2k_{N}(\rho_{N}/\rho_{1})}$, ${\tilde{r}}_{p}^{\left(\beta \right)}=\frac{r_{p}^{\left(\beta \right)}}{r_{p}^{\left(\beta \right)}+r_{d}^{\left(\beta \right)}+\delta^{\left(\beta \right)}+k_{\beta -1}(\rho_{\beta -1}/\rho_{\beta })}$ for 2 ≤ β ≤ N, ${\tilde{k}}_{\beta }^{\left(p\right)}=\frac{k_{\beta }(\rho_{\beta }/\rho_{\beta +1})}{r_{p}^{\left(\beta +1\right)}+r_{d}^{\left(\beta +1\right)}+\delta^{\left(\beta +1\right)}+k_{\beta }(\rho_{\beta }/\rho_{\beta +1})}$ for 1≤ β ≤ N-1, and ${\tilde{k}}_{N}^{\left(p\right)}=\frac{k_{N}(\rho_{N}/\rho_{1})}{r_{p}^{\left(1\right)}+r_{d}^{\left(1\right)}+\delta^{\left(1\right)}+2k_{N}(\rho_{N}/\rho_{1})}$

$R_{\beta \leftarrow \beta^{\prime }}^{\left(p\right)}=\left(\prod_{j=\beta^{\prime }}^{\beta -\text{1}}{\tilde{k}}_{j}^{\left(p\right)}\right){\tilde{r}}_{p}^{\left(\beta^{\prime }\right)}$ and $M_{\beta }^{\left(p\right)}=\left(\prod_{j=\text{1}}^{\beta -\text{1}}{\tilde{k}}_{j}^{\left(p\right)}\right){\tilde{k}}_{N}^{\left(p\right)}$ for 2 ≤ β ≤ N.

### Stochastic simulations

Multi-scale stochastic simulations were performed in MATLAB. The code can be found at https://github.com/indraniny/NK_cell_cycle_and_signaling, and a descriptive illustration is provided in S8 Fig. The simulation was seeded with 400 cells for the linear model (or 20 cells for the non-linear model), initialized with the abundances of each protein and a cell cycle stage designation. See “Initial Condition Generation” in Methods for details. Then, for each cell, a Gillespie simulation is performed [56], where the cell can perform any protein-level reaction, or progress to the next stage of cell cycle. Then, for each cell, a Gillespie simulation is performed [56], where the cell can perform any protein-level reaction, or progress to the next stage of cell cycle. If the cell reaches the final stage (i.e., M stage) and advances, it divides, generating a second daughter cell. Proteins from the parent cell are randomly split between the two daughter cells according to a binomial distribution. Each cell also retains the “time” advanced from the previous timesteps in the Gillespie simulation for that cell. The process continues, with each cell independently selecting reactions to perform via the Gillespie simulation. When a cell is about to complete a reaction that would advance it past the endpoint time of interest, it is removed from the simulation and placed in the output. The simulation continues until all cells have been removed. The resulting output is a collection of cells that would be observed at the endpoint time of interest, originating from the input cells. Finally, we calculated the average behavior of protein abundances from the collections of cells obtained at the endpoint time for each cell cycle stage.

For non-linear simulations that require a volume correction (i.e., for a second-order reaction that governs binding of two proteins), the propensity for the reaction to occur decreases as the volume increases, which is correlated with progression through the cell cycle. To account for this, we assume that the propensity of the reaction to occur in each stage is given by, $k_{i}^{\prime }=\frac{k}{V_{i}}$

*,* where $V_{i}=\text{1},\;\text{1}.\text{3},\;\text{1}.\text{6},\;\text{2}.\text{0}$ represents the volume factors in G1, S, G2, and M, respectively. This allows the volumes of each stage to take values between initial volume and twice the initial volume, mimicking a monotonic increase in cell volume up to a doubling of the cell volume. The simulations for the linear model, shown in Fig 3, took approximately one hour. The simulations for the non-linear model, which produced the results shown in Fig 4, had runtimes ranging from approximately 1–35 hours.

### Generation of initial conditions for non-linear models

For the non-linear model, we began with a population of 20 cells, each containing 80 molecules of the enzyme (E) and 80 molecules of the substrate (A) (Fig 4B-4C, S1 and S2 Tables). Initially, all cells were assumed to be in the G1 stage of the cell cycle. Over time, these cells progress through the subsequent stages-S stage, G2 stage, and M stage before undergoing division to produce two daughter cells. The signaling pathway involves the binding of the enzyme (E) to the substrate (A), forming a complex (EA) that is further phosphorylated to create the product (A*), as described in Fig 4A (details in results section).We then let the system of reactions run for a long period (~ 5 generations of cells or 170 hours) until the mean protein concentrations of all species achieve the steady state. At this point, we calculated the average protein abundances per cell at cell cycle stages G1, S, G2, and M. Because stage M has the shortest duration (~ 1.4 h), the number of cells in this stage is relatively low. In contrast, stage G1 has the longest duration, resulting in a higher number of cells (~14 h) (S1 and S2 Tables). Consequently, the fluctuation in average protein abundance is more pronounced during stage M, while it is less variable in the G1 stage. We independently ran 20 simulation replicates of identical initial configurations to calculate the standard error of average protein abundance (SEM) in Fig 4B-4C.

### Parameter estimation in non-linear signaling models using CyTOF data

To study the variation of phospho-protein intensity across cell cycle stages, we selected pS6 and pAkt (denoted as A* in Fig 4A) from CyTOF measurement. We randomly sampled a small subset of their cell configurations at 2 min as inputs to our non-linear signaling model, which was evolved for an additional 2 min and 6 min to fit the observed non-monotonic (decay-rise) pS6 dynamics at 4 min and monotonic (rise-rise) pAkt dynamics at 8 min, respectively, thereby estimating the phosphorylation rate r_p_ (Fig 4A).

We incorporated cell-to-cell variations in the initial abundances of the enzyme (E), substrate (S), and enzyme-substrate complex (C) by assuming log-normal distributions (Lognormal(μ,σ^2^)) for these abundances with mean μ and standard deviation, σ = 0.05. We assumed the total protein abundances are in the steady state and the signaling reactions evolve with time upon NKG2D stimulation. The ratios of the mean values μ for E, S, and C, at S, G2, and M stages with G1 stage are taken to be the same as the ratios obtained at the steady state (Eqn. 68, S1 Text) in the model when the rates for cell cycle transitions, protein production, and the decay are set to the values shown in the legend of Fig 4D and 4E. The initial number of the mean μ for E, S, and C in the sub-volume (~0.132 μm^S^, S1 Table) within the NK cell cytoplasm at G1 is set to 300 molecules. To estimate the abundances of pS6 or pAkt in the sub-volume in experiments, the fluorescence intensities at 2 min were multiplied by a scaling factor of 100 and floored (i.e., any value <1 is set to 0), while intensities at later time points (4 and 8 min) were scaled similarly without flooring. Log-transformed abundances were then used to compute the mean ($mean_{observed}(i)$) and variance ($\sigma_{observed}^{\text{2}}(i))$ in G1, S, and G2-M stages (cell cycle stage denoted by *i*) from the data at 4 min or 8 min. The log-transformed abundances in the model are used to compute the mean values, $mean_{model}(i)$, in the model.

To estimate the phosphorylation rate r _p_ (Fig 4A) of S6 and Akt under both the neutral and active scenarios, we optimized the residual sum of squares (RSS) by fitting the measured pS6 and pAkt data at 4 min and 8 min,

$RSS=\sum_{i=\text{1}}^{n}\frac{\begin{array}{c} {\left(mean_{observed}(i)-mean_{model}(i)\right)}^{\text{2}}\\ \; \end{array}}{\sigma_{observed}^{\text{2}}(i)}$.

To further selecting model between active and neutral scenario, we computed the Akaike Information Criterion (AIC) [42] score using [57]


$$\begin{array}{c} AIC=n\;\text{log}\left(\frac{RSS}{n}\right)+\text{2}K+\frac{\text{2}K(K+\text{1})}{\left(n-K-\text{1}\right)}. \end{array}$$

Here, n = 3 (G1, S, and G2-M stages) represent the number of data points, and K is the number of estimated parameters. Five model parameters - k_on_, k_off_, r, δ, r_d_ (in Fig 4A) were fixed (see captions of Fig 4D and 4E), while the phosphorylation rate $r_{p}$was estimated. In the neutral scenario, $r_{p}$was assumed to be constant, yielding one parameter, whereas in the active scenario $r_{p}$was allowed to vary across G1, S, and G2 (with M assumed equal to G2), yielding three parameters. The difference in the AIC values (ΔAIC) for the between the active and the neutral case generated values lower than 2 [57] which strongly supports the active case for describing the data.

## Supporting information

S1 Fig UMAP visualization of various markers across cell cycle stages for IL-2 treated NKG2D-stimulated primary CD56^dim^ NK cells. (TIFF)

S2 Fig The mean intensities of various signaling proteins across cell cycle stages for IL2-treated NKG2D-stimulated primary CD56^dim^ NK cells. (TIFF)

S3 Fig Variation of the abundances of signaling proteins in NKG2D-stimulated NKL cells residing in different cell cycle stages. (TIFF)

S4 Fig Variation of the abundances of signaling proteins in Ly49H-stimulated NKL cells residing in different cell cycle stages. (TIFF)

S5 Fig Normalized histograms of the natural log-transformed intensities (I) for the activation markers for IL2-treated NKG2D-stimulated primary CD56^dim^ NK cells. (TIFF)

S6 Fig Degranulation in primary CD56^dim^ NK cells vs. NKL cells. (TIFF)

S7 Fig Sensitivity in variation of the abundances of signaling proteins in NKG2D-stimulated primary CD56^dim^ NK cells and NKL cells residing in different cell cycle stages. (TIFF)

S8 Fig A descriptive illustration of multi-scale stochastic simulation. (TIFF)

S9 Fig Comparison of the mean abundances of the intermediate complex and phosphoprotein (a*) between *in silico* simulation, the approximated formula and exact formula for the non-linear protein model at steady state, related to Fig 4B in the main text. (TIFF)

S10 Fig Variation in the CyTOF intensities of signaling proteins in NKG2D-stimulated primary human CD56^dim^ NK cells across different cell cycle stages for a biological replicate (different donor) than the one presented in Fig 2H (main text). (TIFF)

S11 Fig Distributions of total protein *a* at steady state across cell cycle stages simulated from non-linear model simulations shown in Fig 4B (main text). (TIFF)

S12 Fig Pattens of variations of protein intensities for NKG2D-stimulated primary CD56^dim^ NK cells across cell cycle stages for shifted geometric means. (TIFF)

S1 Table Parameter values used in the non-linear in-silico model (rates used here are from Table 1). (PDF)

S2 Table Model parameters and statistical comparisons for Fig 4. (PDF)

S3 Table Mechanism of protein abundances across cell cycle stages in silico model for realistic parameter ranges given in S1 Table. (PDF)

S4 Table Mass cytometry Ab panel used for analysis of primary NK cells. (PDF)

S5 Table Mass cytometry Ab panel used for analysis of NKL cells. (PDF)

S1 Text This document contains all mathematical derivations and supporting text cited in the main text. Sections 1–4: Derivation of the master equation and the dynamics of relative cell number *ρ*, average protein levels *a* and a* per cell across different cell cycle stages for the linear model presented in Fig 3. Sections 5–8: Steady-state solutions for ρ, *a* and a* in the linear model. Section 9: Minimal model for protein synthesis-degradation dynamics. Section 10: Reaction rates for the zeroth-, first- and second-order re-action within a small simulation volume *V*ϵ. Section 11: Description and approximate steady state analyses of minimal non-linear signaling model shown in Fig 4. Examples for monotonic and non-monotonic variations in complex (C) in steady state of cell cycle transitions. Fold-change calculation of mean phospho-protein levels in an in-silico mode across cell cycle stages. The sensitivity of phosphoprotein variation (monotonic vs. non-monotonic) to model parameters. Calculation of variation in protein abundances for the non-monotonic neutral cases.
(PDF)

## References

[1] Lanier LL. Up on the tightrope: Natural killer cell activation and inhibition. Nat Immunol. 2008;9(5):495–502. doi: 10.1038/ni1581 18425106 PMC2669298

[2] Long EO, Kim HS, Liu D, Peterson ME, Rajagopalan S. Controlling natural killer cell responses: Integration of signals for activation and inhibition. Annu Rev Immunol. 2013;31:227–58. doi: 10.1146/annurev-immunol-020711-075005 23516982 PMC3868343

[3] Abel AM, Yang C, Thakar MS, Malarkannan S. Natural killer cells: Development, maturation, and clinical utilization. Front Immunol. 2018;9:1869. doi: 10.3389/fimmu.2018.01869 30150991 PMC6099181

[4] Mesecke S, Urlaub D, Busch H, Eils R, Watzl C. Integration of activating and inhibitory receptor signaling by regulated phosphorylation of Vav1 in immune cells. Sci Signal. 2011;4(175):ra36. doi: 10.1126/scisignal.2001325 21632469

[5] Grewal RK, Das J. Spatially resolved in silico modeling of NKG2D signaling kinetics suggests a key role of NKG2D and Vav1 Co-clustering in generating natural killer cell activation. PLoS Comput Biol. 2022;18(5):e1010114. doi: 10.1371/journal.pcbi.1010114 35584138 PMC9154193

[6] Millan AJ, et al. Spleen tyrosine kinase (SYK) negatively regulates ITAM-mediated human NK cell signaling and CD19-CAR NK cell efficacy. bioRxiv. 2024. 10.1093/jimmun/vkaf012 PMC11952873 40073103

[7] Milo R, Jorgensen P, Moran U, Weber G, Springer M. BioNumbers--the database of key numbers in molecular and cell biology. Nucleic Acids Res. 2010;38(Database issue):D750-3. doi: 10.1093/nar/gkp889 19854939 PMC2808940

[8] Robertson MJ, Cochran KJ, Cameron C, Le JM, Tantravahi R, Ritz J. Characterization of a cell line, NKL, derived from an aggressive human natural killer cell leukemia. Exp Hematol. 1996;24(3):406–15. 8599969

[9] Alberts B, et al. Essential cell biology. Garland Science. 2015.

[10] Alber AB, Paquet ER, Biserni M, Naef F, Suter DM. Single live cell monitoring of protein turnover reveals intercellular variability and cell-cycle dependence of degradation rates. Mol Cell. 2018;71(6):1079-1091.e9. doi: 10.1016/j.molcel.2018.07.023 30146318

[11] Herr P, Boström J, Rullman E, Rudd SG, Vesterlund M, Lehtiö J, et al. Cell cycle profiling reveals protein oscillation, phosphorylation, and localization dynamics. Mol Cell Proteomics. 2020;19(4):608–23. doi: 10.1074/mcp.RA120.001938 32051232 PMC7124475

[12] Liu P, Begley M, Michowski W, Inuzuka H, Ginzberg M, Gao D, et al. Cell-cycle-regulated activation of Akt kinase by phosphorylation at its carboxyl terminus. Nature. 2014;508(7497):541–5. doi: 10.1038/nature13079 24670654 PMC4076493

[13] Davidson G, Niehrs C. Emerging links between CDK cell cycle regulators and Wnt signaling. Trends Cell Biol. 2010;20(8):453–60. doi: 10.1016/j.tcb.2010.05.002 20627573

[14] Wu J, Song Y, Bakker AB, Bauer S, Spies T, Lanier LL, et al. An activating immunoreceptor complex formed by NKG2D and DAP10. Science. 1999;285(5428):730–2. doi: 10.1126/science.285.5428.730 10426994

[15] Mukherjee S, Jensen H, Stewart W, Stewart D, Ray WC, Chen S-Y, et al. In silico modeling identifies CD45 as a regulator of IL-2 synergy in the NKG2D-mediated activation of immature human NK cells. Sci Signal. 2017;10(485):eaai9062. doi: 10.1126/scisignal.aai9062 28655861 PMC5556952

[16] Herr P, et al. Cell cycle profiling reveals protein oscillation, phosphorylation, and localization dynamics. Mol Cell Proteomics. 2020;19:608–23. doi: 10.1074/mcp.RA120.001938 PMC7124475 32051232

[17] Boström J, Sramkova Z, Salašová A, Johard H, Mahdessian D, Fedr R, et al. Comparative cell cycle transcriptomics reveals synchronization of developmental transcription factor networks in cancer cells. PLoS One. 2017;12(12):e0188772. doi: 10.1371/journal.pone.0188772 29228002 PMC5724894

[18] Lanier LL. NKG2D receptor and its ligands in host defense. Cancer Immunol Res. 2015;3(6):575–82. doi: 10.1158/2326-6066.CIR-15-0098 26041808 PMC4457299

[19] Smith KM, Wu J, Bakker AB, Phillips JH, Lanier LL. Ly-49D and Ly-49H associate with mouse DAP12 and form activating receptors. J Immunol. 1998;161(1):7–10. doi: 10.4049/jimmunol.161.1.7 9647200

[20] Salazar C, Höfer T. Kinetic models of phosphorylation cycles: A systematic approach using the rapid-equilibrium approximation for protein-protein interactions. Biosystems. 2006;83(2–3):195–206. doi: 10.1016/j.biosystems.2005.05.015 16233950

[21] Gardiner CW. Handbook of stochastic methods. Springer Berlin; 1985.

[22] Chao HX, Fakhreddin RI, Shimerov HK, Kedziora KM, Kumar RJ, Perez J, et al. Evidence that the human cell cycle is a series of uncoupled, memoryless phases. Mol Syst Biol. 2019;15(3):e8604. doi: 10.15252/msb.20188604 30886052 PMC6423720

[23] Vittadello ST, McCue SW, Gunasingh G, Haass NK, Simpson MJ. Mathematical models incorporating a multi-stage cell cycle replicate normally-hidden inherent synchronization in cell proliferation. J R Soc Interface. 2019;16(157):20190382. doi: 10.1098/rsif.2019.0382 31431185 PMC6731503

[24] Yates CA, Ford MJ, Mort RL. A Multi-stage representation of cell proliferation as a markov process. Bull Math Biol. 2017;79(12):2905–28. doi: 10.1007/s11538-017-0356-4 29030804 PMC5709504

[25] Perez-Carrasco R, Beentjes C, Grima R. Effects of cell cycle variability on lineage and population measurements of messenger RNA abundance. J R Soc Interface. 2020;17(168):20200360. doi: 10.1098/rsif.2020.0360 32634365 PMC7423421

[26] Jia C, Singh A, Grima R. Concentration fluctuations in growing and dividing cells: Insights into the emergence of concentration homeostasis. PLoS Comput Biol. 2022;18(10):e1010574. doi: 10.1371/journal.pcbi.1010574 36194626 PMC9565450

[27] Jun S, Taheri-Araghi S. Cell-size maintenance: Universal strategy revealed. Trends Microbiol. 2015;23(1):4–6. doi: 10.1016/j.tim.2014.12.001 25497321

[28] Xie S, Swaffer M, Skotheim JM. Eukaryotic cell size control and its relation to biosynthesis and senescence. Annu Rev Cell Dev Biol. 2022;38:291–319. doi: 10.1146/annurev-cellbio-120219-040142 35562854

[29] Lin J, Amir A. Homeostasis of protein and mRNA concentrations in growing cells. Nat Commun. 2018;9(1):4496. doi: 10.1038/s41467-018-06714-z 30374016 PMC6206055

[30] Chen Y, Zhao G, Zahumensky J, Honey S, Futcher B. Differential scaling of gene expression with cell size may explain size control in budding yeast. Mol Cell. 2020;78(2):359-370.e6. doi: 10.1016/j.molcel.2020.03.012 32246903 PMC7963363

[31] Lanz MC, Zatulovskiy E, Swaffer MP, Zhang L, Ilerten I, Zhang S, et al. Increasing cell size remodels the proteome and promotes senescence. Mol Cell. 2022;82(17):3255-3269.e8. doi: 10.1016/j.molcel.2022.07.017 35987199 PMC9444988

[32] Golding I, Paulsson J, Zawilski SM, Cox EC. Real-time kinetics of gene activity in individual bacteria. Cell. 2005;123(6):1025–36. doi: 10.1016/j.cell.2005.09.031 16360033

[33] Huh D, Paulsson J. Random partitioning of molecules at cell division. Proc Natl Acad Sci U S A. 2011;108(36):15004–9. doi: 10.1073/pnas.1013171108 21873252 PMC3169110

[34] Chatterjee M, Acar M. Heritable stress response dynamics revealed by single-cell genealogy. Sci Adv. 2018;4(4):e1701775. doi: 10.1126/sciadv.1701775 29675464 PMC5906080

[35] Chylek LA, Akimov V, Dengjel J, Rigbolt KTG, Hu B, Hlavacek WS, et al. Phosphorylation site dynamics of early T-cell receptor signaling. PLoS One. 2014;9(8):e104240. doi: 10.1371/journal.pone.0104240 25147952 PMC4141737

[36] Hui E, Vale RD. In vitro membrane reconstitution of the T-cell receptor proximal signaling network. Nat Struct Mol Biol. 2014;21(2):133–42. doi: 10.1038/nsmb.2762 24463463 PMC4062301

[37] Milo R, Phillips R. Cell biology by the numbers. Garland Science; 2015.

[38] Trausch-Azar J, Leone TC, Kelly DP, Schwartz AL. Ubiquitin proteasome-dependent degradation of the transcriptional coactivator PGC-1{alpha} via the N-terminal pathway. J Biol Chem. 2010;285(51):40192–200. doi: 10.1074/jbc.M110.131615 20713359 PMC3001001

[39] Nayak I, et al. Modeling the response to interleukin‐21 to inform natural killer cell immunotherapy. Immunology and Cell Biology. 2025;103:192–212. 10.1111/imcb.12848 PMC11792776 39865344

[40] Denman CJ, Senyukov VV, Somanchi SS, Phatarpekar PV, Kopp LM, Johnson JL, et al. Membrane-bound IL-21 promotes sustained ex vivo proliferation of human natural killer cells. PLoS One. 2012;7(1):e30264. doi: 10.1371/journal.pone.0030264 22279576 PMC3261192

[41] Murray JD. Mathematical biology. I, Introduction. Springer; 2002.

[42] Akaike H. International encyclopedia of statistical science. Springer. 2025. p. 41–2.

[43] Stonyte V, Boye E, Grallert B. Regulation of global translation during the cell cycle. J Cell Sci. 2018;131(17):jcs220327. doi: 10.1242/jcs.220327 30072440

[44] Lewis DA, Ly T. Cell cycle entry control in naïve and memory CD8+ T cells. Front Cell Dev Biol. 2021;9:727441. doi: 10.3389/fcell.2021.727441 34692683 PMC8526999

[45] Das J, Ho M, Zikherman J, Govern C, Yang M, Weiss A, et al. Digital signaling and hysteresis characterize ras activation in lymphoid cells. Cell. 2009;136(2):337–51. doi: 10.1016/j.cell.2008.11.051 19167334 PMC2662698

[46] Schwabe A, Bruggeman FJ. Contributions of cell growth and biochemical reactions to nongenetic variability of cells. Biophys J. 2014;107(2):301–13. doi: 10.1016/j.bpj.2014.05.004 25028872 PMC4104058

[47] Soltani M, Vargas-Garcia CA, Antunes D, Singh A. Intercellular variability in protein levels from stochastic expression and noisy cell cycle processes. PLoS Comput Biol. 2016;12(8):e1004972. doi: 10.1371/journal.pcbi.1004972 27536771 PMC4990281

[48] Wang Y, Yu Z, Grima R, Cao Z. Exact solution of a three-stage model of stochastic gene expression including cell-cycle dynamics. J Chem Phys. 2023;159(22):224102. doi: 10.1063/5.0173742 38063222

[49] Dessalles R, Fromion V, Robert P. Models of protein production along the cell cycle: An investigation of possible sources of noise. PLoS One. 2020;15(1):e0226016. doi: 10.1371/journal.pone.0226016 31945071 PMC6964835

[50] Rega C, Tsitsa I, Roumeliotis TI, Krystkowiak I, Portillo M, Yu L, et al. High resolution profiling of cell cycle-dependent protein and phosphorylation abundance changes in non-transformed cells. Nat Commun. 2025;16(1):2579. doi: 10.1038/s41467-025-57537-8 40089461 PMC11910661

[51] Mukherjee S, Jensen H, Stewart W, Stewart D, Ray WC, Chen S-Y, et al. In silico modeling identifies CD45 as a regulator of IL-2 synergy in the NKG2D-mediated activation of immature human NK cells. Sci Signal. 2017;10(485):eaai9062. doi: 10.1126/scisignal.aai9062 28655861 PMC5556952

[52] Jensen H, Chen S-Y, Folkersen L, Nolan GP, Lanier LL. EBI3 regulates the NK cell response to mouse cytomegalovirus infection. Proc Natl Acad Sci U S A. 2017;114(7):1625–30. doi: 10.1073/pnas.1700231114 28143936 PMC5321027

[53] Behbehani GK. Cell cycle analysis by mass cytometry. Cellular quiescence: Methods and protocols. 2018. p. 105–24. 10.1007/978-1-4939-7371-2_8 29030816

[54] Tibshirani RJ, Efron B. An introduction to the bootstrap. Monographs on Statistics and Applied Probability. 1993;57:1–436.

[55] Gardiner CW. Handbook of stochastic methods-for physics, chemistry and the natural sciences. 1985.

[56] Gillespie DT. Exact stochastic simulation of coupled chemical reactions. The Journal of Physical Chemistry. 1977;81:2340–61.

[57] Burnham KP, Anderson DR, Huyvaert KP. AIC model selection and multimodel inference in behavioral ecology: Some background, observations, and comparisons. Behav Ecol Sociobiol. 2010;65(1):23–35. doi: 10.1007/s00265-010-1029-6

[58] Cox C. Delta method. Encyclopedia of biostatistics. 2005.

[59] Berger VW, Zhou Y. Kolmogorov–smirnov test: Overview. 2014.

[60] Weisstein EW. Bonferroni correction. https://mathworld.wolfram.com/. 2004.

[61] Liu Y, Wu H-W, Sheard MA, Sposto R, Somanchi SS, Cooper LJN, et al. Growth and activation of natural killer cells ex vivo from children with neuroblastoma for adoptive cell therapy. Clin Cancer Res. 2013;19(8):2132–43. doi: 10.1158/1078-0432.CCR-12-1243 23378384 PMC3658308

[62] Marchingo JM, Cantrell DA. Protein synthesis, degradation, and energy metabolism in T cell immunity. Cell Mol Immunol. 2022;19(3):303–15. doi: 10.1038/s41423-021-00792-8 34983947 PMC8891282

[63] Wolf T, Jin W, Zoppi G, Vogel IA, Akhmedov M, Bleck CKE, et al. Dynamics in protein translation sustaining T cell preparedness. Nat Immunol. 2020;21(8):927–37. doi: 10.1038/s41590-020-0714-5 32632289 PMC7610365

[64] Eden E, Geva-Zatorsky N, Issaeva I, Cohen A, Dekel E, Danon T, et al. Proteome half-life dynamics in living human cells. Science. 2011;331(6018):764–8. doi: 10.1126/science.1199784 21233346

[65] Schreiber G, Haran G, Zhou H-X. Fundamental aspects of protein-protein association kinetics. Chem Rev. 2009;109(3):839–60. doi: 10.1021/cr800373w 19196002 PMC2880639

[66] Bu JY, Shaw AS, Chan AC. Analysis of the interaction of ZAP-70 and syk protein-tyrosine kinases with the T-cell antigen receptor by plasmon resonance. Proc Natl Acad Sci U S A. 1995;92(11):5106–10. doi: 10.1073/pnas.92.11.5106 7761456 PMC41857

[67] Clemens L, Kutuzov M, Bayer KV, Goyette J, Allard J, Dushek O. Determination of the molecular reach of the protein tyrosine phosphatase SHP-1. Biophys J. 2021;120(10):2054–66. doi: 10.1016/j.bpj.2021.03.019 33781765 PMC8204385

