# Supplementary material for: Natural killer cell receptor signaling and activation depend on cell cycle stages
Source: PLoS Comput Biol. 2026 Oct 13;22(10):e1014825. doi: 10.1371/journal.pcbi.1014825 (PMC13705607; doi:10.1371/journal.pcbi.1014825)
Supplement: Supplementary file 1 [file pcbi.1014825.s013.pdf]

Table S1: Parameter values used in the non-linear in-silico model (rates used here are from Table 1)

Parameters used in simulation	Parameter values used in the simulation	Remarks
Enzyme (E)	80 molecules	Assumption. The cytosolic volume (V_{cyto}) of NK cell is ~ 80 um^3 assuming cell and nucleus radii to be 3 um and 2 ums, respectively. For simulation, we assumed a fraction of cytosolic cell volume as a cell to reduce computational cost.
Substrate (A)	80 molecules	Assumption. The cytosolic volume of NK cell. V_{cyto} is ~ 80 um^3 assuming cell and nucleus radii to be 3 um and 2 ums, respectively. For simulation, we assumed a fraction of cytosolic cell volume as a cell to reduce computational cost.
Cell volume (V_e)	0.132 um^3	Since inside the cell the protein density is ~1 uM or 602 molecules per um^3 (e.g., ZAP70 density in NK cell), the simulation volume (V_e) to accommodate 80 copies is ~ 0.132 um^3.
Cell cycle transition rate	[0.07, 0.1, 0.2, 0.7] h^{-1}	For primary NK cell, the cell cycle takes around 30 hrs. How the time durations are distributed into G1, S, G2 and M are not known. We have calculated the cell cycle transition rates in primary NK cell using the proportion of duration of cell cycle stages in Hela cell.
Simulation time	150 h -170 h	Assuming avg. protein per cell will reach to a steady state value after 170 hours or ~ 5 complete cycles.

Enzyme or Substrate synthesis rate (r)	18-5350 molecules h^{-1}	This is a zeroth order reaction rule. Because we have considered a fraction of cell volume for simulation, we scaled the realistic synthesis rate (10800-3240000 copies per hour) from literature by a multiplicative factor (V_e/V_{cyto}). The calculation of multiplicative factor is shown in **S1 Text,** section 10.
Protein degradation rate (δ)	0.03-0.9 h^{-1}	This is a first-order reaction. Thus, it remains same for any cell volume.
Complex formation rate (k_{on})	4.53-4530 h^{-1} per molecule	This is a second order reaction rule. The association range could be 10^5-10^8 M^{-1} s^{-1} . For example, ZAP70 binds to ITAM with rate 5 uM^{-1} s^{-1} = 29.90 μm^3 h^{-1} per copy. Because we have considered a fraction of cell volume for simulation, we scaled the realistic k_{on} from literature by a multiplicative factor ($1/ V_e$) to calculate the propensity which gives 226 h^{-1} per copy. The calculation of multiplicative factor is shown in **S1 Text,** section 10.
Complex dissociation rate (k_{off})	450-7200 h^{-1}	In T cell, ZAP70 unbinding rate from ITAM 0.125 s^{-1}. SHP1 unbinding rate from ITAM 2 s^{-1}.
Protein phosphorylation rate (r_p)	36-7200000 h^{-1}	This is a first-order reaction. Thus, it remains same for any cell volume.
Protein dephosphorylation rate (r_d)	36-7200000 h^{-1}	This is a first-order reaction. Thus, it remains same for any cell volume.
