# Supplementary material for: Natural killer cell receptor signaling and activation depend on cell cycle stages
Source: PLoS Comput Biol. 2026 Oct 13;22(10):e1014825. doi: 10.1371/journal.pcbi.1014825 (PMC13705607; doi:10.1371/journal.pcbi.1014825)
Supplement: Supplementary file 2 [file pcbi.1014825.s014.pdf]

S2 Table. Model parameters and statistical comparisons for Figure 4.

A: Parameter values used in Figure 4B-4C

Figure index	r (molecules h^{-1})	δ (h^{-1})	k_{on} (h^{-1} per molecule)	k_{off} (h^{-1})	r_p (h^{-1})	r_d (h^{-1})	Volume factor at G1, S, G2, M
**B (left)**	18	0.05	15	3000	5000	36	[1, 1.3, 1.6, 2]
**B (middle)**	20	0.5	10	3000	10000	36	[1, 1.3, 1.6, 2]
**B (right)**	20	0.9	5	5000	10000	36	[1, 1.3, 1.6, 2]
**C (left)**	18	0.05	15	3000	[5000, 50, 5000, 5000]	36	[1, 1.3, 1.6, 2]
**C (right)**	18	0.05	15	3000	[5000, 5000, 50, 50]	36	[1, 1.3, 1.6, 2]

Other common parameters for Figures 4B-4C

Enzyme (molecules per cell)	Substrate (molecules per cell)	Cell transition rates (h^{-1})	Simulation time (h)	Cell volume (μm^3)
80	80	[0.07, 0.1, 0.2, 0.7]	150-170	0.132

B: *P*-values are tabulated for *t*-tests to compare the avg. protein abundances between cell cycle stages in Figure 4

	G1-S	S-G2	G2-M
**Fig. B (left)**	1.14×10^{-31}	2.32×10^{-16}	0.04
**Fig. B (middle)**	3.21×10^{-17}	2.19×10^{-6}	8.20×10^{-6}
**Fig. B (right)**	5.73×10^{-10}	2.87×10^{-14}	6.04×10^{-9}
**Fig. C (left)**	1.37×10^{-48}	1.97×10^{-45}	3.04×10^{-4}
**Fig. C (right)**	5.26×10^{-34}	1.78×10^{-47}	2.2×10^{-8}

C: Parameter ranges used to obtain protein variation for sensitivity analysis

r (molecules h^{-1})	δ (h^{-1})	k_{on} (h^{-1} per molecule)	k_{off} (h^{-1})	r_p (h^{-1})	r_d (h^{-1})	Volume factor at G1, S, G2, M
20	[0.03,0.9]	[5,100]	3000	10000	36	[1, 1.3, 1.6, 2]

*parameter values are chosen randomly within the range of δ and k_{on}.
