# Supplementary material for: Natural killer cell receptor signaling and activation depend on cell cycle stages
Source: PLoS Comput Biol. 2026 Oct 13;22(10):e1014825. doi: 10.1371/journal.pcbi.1014825 (PMC13705607; doi:10.1371/journal.pcbi.1014825)
Supplement: Supplementary file 3 [file pcbi.1014825.s015.pdf]

Table S3: Mechanism of protein abundances across cell cycle stages in silico model for realistic parameter ranges given in Table S1

Assuming cell cycle transition rates k_β depends on the cell cycle stage β. See the approximated calculation in **S1 Text,** section 11.

**Protein Behavior G1→ S → G2**	***Neutral* Case with const. signaling rates**	**Condition**	**Protein behavior along G1 →S →G2**			
			**E_{tot}**	**A_{tot}**	**C**	**P**
Monotonic increase (increase, increase)	$r, \delta, k_{on}, k_{off}, r_p, r_d$	$\delta < k_\beta$	Monotonic rise	Monotonic rise	Monotonic rise	Monotonic rise
Non-monotonic (increase, decrease)		x small, Competing $(f_1 \times f_2)$ Where f_1, f_2 are increasing and decreasing functions, respectively.	Monotonic rise	Monotonic rise	Non-monotonic peak	Non-monotonic peak
Monotonic decrease (decrease, decrease)		x small, $\delta \gg k_\beta$ Competing $(f_1 \times f_2)$ Where f_1, f_2 are constant and decreasing functions, respectively.	No change	No change	Monotonic decay	Monotonic decay
Unchanged (no-change, no-change)		$\delta \gg k_\beta$ $K_D' \ll 1$	No change	No change	No change	No change

$K_D' = \frac{K_{off}+\delta+r_p}{K_{on}}$, $E_{tot} = A_{tot} = \frac{r}{\delta}$ = total concentrations of E and A

$f_1 = E_{tot} \times A_{tot}$, f_1 is an increasing or constant function with cell cycle stages.

$f_2 = \frac{1}{A_{tot}+\beta E_{tot}+K_D'}$, f_2 is a decreasing function as cell volume grows with cell cycle stages.

$\beta = 1 + \frac{r_p}{\delta+r_d}$, range $[1, \infty]$

$x = 4\beta f_1 f_2^2$
