# Supplementary material for: Natural killer cell receptor signaling and activation depend on cell cycle stages
Source: PLoS Comput Biol. 2026 Oct 13;22(10):e1014825. doi: 10.1371/journal.pcbi.1014825 (PMC13705607; doi:10.1371/journal.pcbi.1014825)
Supplement: Supplementary file 4 [file pcbi.1014825.s016.pdf]

Table S4: Mass cytometry Ab panel used for analysis of primary NK cells

	Antibody marker	Metal	Ab Clone	Vendor
Surface	CD7	113In	CD7-6B7	BioLegend
	CD45	115In	HI30	BioLegend
	CD57	139La	HCD57	BioLegend
	CD3+CD235+CD61+CD15	141Pr	UCHT1+HIR2+VI-PL2+W6D3	BioLegend
	CD96	142Nd	NK92.39	BioLegend
	CD8	147Sm	RTA-T8	BioLegend
	CD16	148Nd	3G8	Fluidigm
	CD107a	151Eu	H4A3	Fluidigm
	NKG2D	154Sm	1D11	BioLegend
	CRACC	155Gd	162.1	BioLegend
	CD161	158Gd	191B8	Beckman Coulter
	CD11c	159Tb	Bu15	Fluidigm
	2B4	160Gb	PP35	eBioscience
	CD69	162Dy	FN50	Fluidigm
	NKG2A	174Yb	Z199	Beckman Coulter
	CD56	176Yb	NCAM16.2	BD
Intracellular	pCrkL(Y207)	143Nd	Polyclonal	Fluidigm
	pHistone H3(S28)	145Nd	HTA28	BioLegend
	pPLCγ2(Y759)	146Nd	K86-689.37	BD
	pCreb(S133)	149Sm	87G3	Cell Signaling
	pSTAT5(Y694)	150Nd	47	BD
	pAkt(S473)	152Sm	D9E	Fluidigm
	pMAPKAPK2(T334)	153Eu	27B7	Cell Signaling
	Cyclin B	156Gd	GNS-1	BD
	SAP	157Gd	XLP-1D12	Cell Signaling
	DAP12	161Dy	406288	R&D Systems
	EAT2	163Dy	Polyclonal	Abgent
	pSLP76(Y128)	164Dy	J141-668.36.58	BD
	pNFκB(S529)	165Ho	K10-895.12.50	BD
	pRb(S807/S811)	166Er	J112-906	BD
	pErk1/2(T202/Y204)	167Er	D13-14-4E	Fluidigm
	pPlk1(T210)	168Er	K50-483	BD
	p38(T180/Y182)	169Tm	36/p38	BD
	pLAT(Y226)	170Er	J96-1238.58.93	BD
	pZAP70(Y319/Y352)	171Yb	17a	Fluidigm

	pS6(S235/S236)	172Yb	N7-548	Fluidigm
	Ki67	173Yb	SolA15	eBioscience
	pVav1(Y160)	175Lu	Polyclonal	Life Technology
