# Supplementary material for: Natural killer cell receptor signaling and activation depend on cell cycle stages
Source: PLoS Comput Biol. 2026 Oct 13;22(10):e1014825. doi: 10.1371/journal.pcbi.1014825 (PMC13705607; doi:10.1371/journal.pcbi.1014825)
Supplement: Supplementary file 5 [file pcbi.1014825.s017.pdf]

Table S5: Mass cytometry Ab panel used for analysis of NKL cells

	Antibody marker	Metal	Ab Clone	Vendor
Surface	CD45	115In	HI30	BioLegend
	CD11a	139La	HI111	BD
	CD96	142Nd	NK92.39	BioLegend
	CD94	146Nd	DX22	BioLegend
	DNAM1	149Sm	11A8	BioLegend
	CD107a	151Eu	H4A3	Fluidigm
	NKG2D	154Sm	1D11	BioLegend
	CRACC	155Gd	162.1	BioLegend
	CCR7	159Tb	150503	R&D Systems
	2B4	160Gb	PP35	eBioscience
	CD69	162Dy	FN50	Fluidigm
	CD25	169Tm	BC96	BioLegend
	NKG2A	174Yb	Z199	Beckman Coulter
Intracellular	pSHP2(Y580)	141Pr	polycloncal	Cell Signaling
	pSTAT1(Y701)	143Nd	4a	BD
	pSrc(Y418)	144Nd	K98-37	BD
	pAMPK(T172)	145Nd	40H9	Cell Signaling
	pGSK-3a/b(S21/9)	147Sm	37F11	Cell Signaling
	pSTAT5(Y694)	150Nd	47	BD
	pAkt(S473)	152Sm	D9E	Fluidigm
	pMAPKAPK2(T334)	153Eu	27B7	Cell Signaling
	p38(T180/Y182)	156Gd	36/p38	BD
	SAP	157Gd	XLP-1D12	Cell Signaling
	Ki67	158Gd	SolA15	eBioscience
	DAP12	161Dy	406288	R&D Systems
	EAT2	163Dy	Polyclonal	Abgent
	Cyclin B	164Dy	GNS-1	BD
	pNFκB(S529)	165Ho	K10-895.12.50	BD
	pRb(S807/S811)	166Er	J112-906	BD
	pErk1/2(T202/Y204)	167Er	D13-14-4E	Fluidigm
	pHistone H3(S28)	168Er	HTA28	BioLegend
	pMARCKS(S152/56)	170Er	polyclonal	Cell Signaling
	p120 catenin(T916)	171Yb	1/catenin	BD
	pS6(S235/S236)	172Yb	N7-548	Fluidigm
	cPARP	173Yb	F21-852	BD
	pVav1(Y160)	175Lu	Polyclonal	Life Technology

	pCreb(S133)	176Yb	87G3	Cell Signaling
