# Supplementary material for: Natural killer cell receptor signaling and activation depend on cell cycle stages
Source: PLoS Comput Biol. 2026 Oct 13;22(10):e1014825. doi: 10.1371/journal.pcbi.1014825 (PMC13705607; doi:10.1371/journal.pcbi.1014825)
Supplement: Supplementary file 8 [file pcbi.1014825.s020.docx]

Dear Editor,

Thank you for giving us the opportunity to revise our manuscript. We also thank all the three reviewers for constructive and insightful comments. We have substantially revised the manuscript text and added new figures in the main text and the supplementary material to address the reviewers’ comments. A point-by-point response to the reviewers’ comments is appended below. We believe the edits have improved the manuscript and hope its suitable for publication in PLOS Computational Biology.

Sincerely,

Lewis L Lanier

Jayajit Das

---------------------------------- Point-by-point response to reviewers comments--------------------

**Reviewers' comments:**

Reviewer's Responses to Questions

*Reviewer #1: The authors have improved and clarified some of the major concerns.*

*Overall, the manuscript could profit from a cleaner structure, especially in the first part, clearer take-away messages from the different sections and a better discussion of the mathematical model within the discussion section, which is currently missing completely.*

We thank the reviewer for the constructive feedback. In response, we have revised the manuscript to improve the overall structure, particularly in the first part of the paper. We have also added a key take-away paragraph at the end of the subsections within the Results section and expanded the Discussion to include a more detailed discussion of the mathematical model. The revised text is highlighted in the marked version of the manuscript.

*Reviewer #2: Thank you to the authors for clarifying some of my concerns. However, my major concern does not relate to the relative time scales of treatment versus total NK cell cycle duration. I instead remain unconvinced that tracking the “tonicity” of protein abundance at given treatment times across cell cycle phases is an appropriate methodology. I agree with the authors that differences in relative abundances of enzymes as cells progress through the cell cycle could generate different signaling responses depending on which phase of the cell cycle a given cell is in at the time of treatment. The data in Fig 2G demonstrate that the time course of CD69 abundance after stimulation is indeed quite different in S phase compared to G1 or G2/M. In fact, I think that the data in Fig 2G provides a much better representation of the authors’ primary question in this manuscript than the classification of tonicity shown in Fig 2E. In the case of CD69, Fig 2G demonstrates the aforementioned difference in dynamics in S phase, but it also shows that CD69 is always highest in G2/M–an important aspect of the cell cycle-dependence of CD69 abundance that is obfuscated in cthe G1-high->S-low->G2/M-high representation of tonicity at the 32, 64, and 256 min time points. As I stated previously, the CD69 abundance after 32 minutes of stimulation in S phase has no direct temporal relationship to the 32 min time point in G1 or G2/M, so this representation is unnecessarily confusing. I would highly recommend omitting all the language related to tonicity and instead present the data for all proteins as in Fig 2G (or something similar), which preserves the representation of the dynamics within each phase while also allowing a quantitative comparison between phases. I believe that this would greatly improve this manuscript’s interpretability and capture the main message with greater clarity.*

*One last point that should be addressed is that in the longer treatment durations–particularly the 256 min time point–there will be a significant proportion of cells that were in a previous cell cycle phase at the start of treatment than at the time of fixation when the authors assigned cell cycle phase to each cell. For example, a cell in late G1 at the time of treatment will likely be in early S phase after 256 min of stimulation. This is another reason why comparing time points across cell cycle phases is not appropriate. Furthermore, given that overall the ‘non-monotonic’ conditions are enriched in these later time points, this concern directly impacts some of the main claims in the manuscript. At the very least, the authors should consider removing the 256 min time point and sticking with treatment times less than ~1 hour to minimize the impact of this confounder.*

In response to the above suggestion, we have revised the abstract and the text describing the Abstract, Results, and the Discussion sections. In addition, we have added the time-course dynamics of protein intensities within and across the cell-cycle stages in revised Figure 2 and Supplementary Figures 2-4. We have also added a schematic diagram (Figure 2G) to explain the ‘tonicity’ analysis. The revised text is highlighted in the marked version (gray) of the manuscript.

*Reviewer #3: The authors have done substantial changes to the manuscript which address all the questions that I initially had.*

We thank the reviewer and are pleased that the revisions addressed the initial concerns.
