# Supplementary material for: Natural killer cell receptor signaling and activation depend on cell cycle stages
Source: PLoS Comput Biol. 2026 Oct 13;22(10):e1014825. doi: 10.1371/journal.pcbi.1014825 (PMC13705607; doi:10.1371/journal.pcbi.1014825)
Supplement: Supplementary file 9 [file pcbi.1014825.s019.docx]

**Manuscript number:** RC-2025-02963R

**Corresponding author(s):** Jayajit, Das

*[Please use this template only if the submitted manuscript should be considered by the affiliate journal as a full revision in response to the points raised by the reviewers.*

*If you wish to submit a preliminary revision with a revision plan, please use our "*[*Revision Plan*](https://docs.google.com/document/u/0/d/1HHp62Tfeb44nd3Gu_3ChB0b8gmMdEog7GjZcZFwHSiQ/edit)*" template.* ***It is important to use the appropriate template to clearly inform the editors of your intentions.****]*

We thank all the reviewers for constructive suggestions and insightful comments. We have performed additional simulations and data analyses to address these comments, and included these results in the revised main text, main text figures, and the supplementary material.

## Point-by-point description of the revisions

*1. Reviewer #1 (Evidence, reproducibility and clarity (Required)):*

*Summary: The paper reports the results of a study that examines how cell cycle stages*

*influence NK cell receptor signaling. The authors find that while most signaling proteins*

*increase monotonically with cell cycle progression, a subset shows non-monotonic*

*variations. Simple computational models are used to explore mechanisms to qualitatively explain the observations.*

*Major comments:* 
*1. I am not convinced that the use of models here substantially contributes to the understanding of the observations. The reason is that the results are fairly intuitive and actually to a good extent already well known to those who construct models of reaction kinetics including cell-cycle dynamics. So for example the observation that protein numbers increase mononotically with cell-cycle progression is the obvious thing to expect because since most proteins have a lifetime that exceeds the mean cell-cycle duration then it follows that naturally the protein numbers have to increase during the cell-cycle. This already explains the bulk of the observations. For those proteins where there is non-monotic behaviour, indeed there is something more complex going on but here there are many possibilities. As they say, if we have a 2nd order reaction then the firing rate of bimolecular reactions could increase or decrease with cell-cycle progression because it decreases with cell volume and increases with abundance, both of which factors vary with cell-cycle progression. A model is not quite needed to see that this may lead to non-monotic behaviour.* *If the model was fitted to the data, i.e. the experimental distributions of protein abundance with cell-cycle progression were fitted to the model and then these are used to constrain possible mechanisms, then yes I would agree that the model brings in some added benefit.*

We agree that the monotonic increases in protein abundances are intuitive. The model helped to quantify the changes in the protein abundances of signaling proteins under different modes of cell cycle regulation (neutral and active cases). However, to address the above comment, we used the model to glean additional mechanisms. We fitted the signaling kinetics of two proteins, pS6 and pAkt. pS6 intensities at t=4 min post-NKG2D stimulation showed non-monotonic variations and pAkt intensities at t=8 min post-NKG2D stimulation showed monotonic increases for G1→ S→ G2-M in primary CD56^dim^ NK cells in our CyTOF measurements. Our comparison of models describing the neutral case and the active case using AIC-based model selection showed the active case better describes the signaling kinetics for both proteins, including pAkt that showed the intuitive monotonic increase across cell cycle stages. Thus, the application of the model to the data suggests the signaling kinetics are likely to be regulated actively by cell cycle processes even for monotonic changes of protein abundances. We have revised the main text, methods, the figures, and the supplementary material to discuss the results. Below we append the text (Results section, page 14) and the figures (Figs. 4D and 4E) we included in the manuscript describing the above results for convenience of the reviewer.

Next, we evaluated whether the protein signaling kinetics in NK cells as measured in our CyTOF experiments are described more preferably by active or neutral case. We chose two types of signaling kinetic patterns: (1) average pS6 shows a non-monotonic behavior; a decrease for G1→ S followed by an increase for S→ G2-M at t=4 min post-NKG2D stimulation and (2) average pAkt increases monotonically from G1→ S→ G2-M at t=8 min post-NKG2D stimulation. We used a non-linear enzymatic phosphorylation model (**Figure 4A**) for pS6 and pAkt, where the phosphorylation rate, r_p_, is the same (neutral case) or can vary (active case) across cell cycle stages. The model is simulated for a small sub-volume (~ 0.132 μm^3^, **Supplementary Table S1**) within the cell where the reactants can be well mixed. Using the distributions of pS6 and pAkt measured in CyTOF at t=2 min as initial distributions of the number of protein molecules for the product (A*) within the sub-volume, we evolved the abundances of pS6 and pAkt in individual cells until t=4 min and t=8 min, respectively, using the model for the neutral and active cases (see Methods). The measured abundances of pS6 at 4 min and pAkt at 8 min in the CyTOF experiments were used to estimate the rate constant(s) (r_p_ in the neutral case, and three cell cycle stage-dependent r_p_ values in the active case) by minimizing the residual sum of squares (RSS) between the model and the data for the arithmetic mean of the log of the abundances (or the log of the geometric mean). Using Akaike Information Criterion (AIC)^1^ (see Methods), an approach for model selection, we found that the active scenario described the measured data at t=4 min and 8 min better (ΔAIC = -32 for pS6 and -47 for pAkt) compared to the neutral scenario for both signaling proteins. Therefore, both for monotonic and non-monotonic changes across cell cycle stages, the cell cycle-dependent phosphorylation of these signaling proteins is more consistent with the data.

**Figure 4D and 4E: (D)** The mean of logarithmic_10_ abundances of pS6 at G1, S, and G2-M stages are shown for experiment (blue bar) and model (red bar) at 4 min. (Left) Shows the model fit for the neutral case with a single parameter is used for the fitting (best fit estimate,$\hat{r_{p}}$=456.0 hr^-1^). (Right) Shows the model fit for the active case with four different r_p_ fitting parameters, describing the phosphorylation in different cell cycle stages. The best fit estimates are $\hat{r_{p}}$= [254.0, 87.0, 1306.0, 1306.0] hr^-1^ at cell cycle stages G1, S, G2 and M, respectively. **(E)** The mean of logarithmic abundances of pAkt at G1, S, and G2-M stages are shown for experiment (blue bar) and model (red bar) at 8 min. (Left) Shows the model fit for the neutral case with a single parameter $\hat{r_{p}}$= 1464.0 hr^-1^ or the active case (right) with four fitting parameters denoting r_p_ at different cell cycle stages. The best fit estimate is given by $\hat{r_{p}}$= [416.0, 534.0, 1822.0, 1822.0] hr^-1^ at cell cycle stages G1, S, G2 and M, respectively. **(D-E)** Error bars represent the standard error of means computed using the of logarithmic of protein abundances. Parameters used in the model are r=5350 molecules hr^-1^, δ=0.03 hr^-1^, k_on_=4500 hr^-1^ molecule^-1^, k_off_ = 450.0 hr^-1^, r_d_= 2500 hr^-1^ and $k_{\beta}$ = [0.07, 0.1, 0.2, 0.7] hr^-1^ from Supplementary Table S1.

*2. Another criticism is the modelling approach itself involves strong simplifications that may not be entirely realistic."*

We thank the reviewer for the detailed comments below. In this study, we focused on the effect of cell cycle transitions on NK cell signaling that occurs in a much faster time scale (~minutes) compared to the NK cell division times (~30 hours) - thus during cell signaling, a single NK cell is less likely to undergo multiple cell divisions. Therefore, we considered a simplified model where cell volume dependent transition rates or protein production rates used for cell cycle models describing various paradigms (e.g., sizer, adder, timer) of cell size homeostasis are not explicitly considered.

*(i) the volume does not seem to change within one cell-cycle stage, e.g. it is 1.3 for all times within the S phase.. "This assumption may be questionable, particularly for cell-cycle stages that occupy a large portion of the cycle." The cell volume generally should vary continuously with time within the cell-cycle and because the propensities are time-dependent then the SSA is not anymore exact and hence one needs to use modifications of it which account for such phenomena.*

We reasoned that cell volume changes much more slowly (over several hours) than the signaling dynamics (occurs in minutes, at least 50-fold faster than the average durations of the cell cycle stages G1, S, G2 and M, See Table 1). Thus, within the short time scales of signaling considered here, the cell volume remains approximately constant in our SSA model, which we believe to be a good approximation.

*(ii) the doubling of gene copy number due to DNA replication seems to have been omitted from the model. This is expected to lead to a considerable change in the protein numbers at the point in the cell-cycle where DNA replication occurs and hence appears to be an important factor for this study.*

We did not explicitly model doubling of DNA or gene transcription for simplicity. However, the effects of these cell cycle stage-dependent processes can be implemented implicitly as cell cycle stage-specific protein production/degradation rates in our model (Figure 4A).

*(iii) how do we reconcile protein concentration homeostasis with the models described in this paper? This is a well-known phenomenon, see for e.g. Nature communications 9.1 (2018): 4496 and references therein.*

The concentration homeostasis with cell size for mRNA/proteins have been usually observed for the total mRNA/protein content, and individual mRNA^2^ or protein^3^ concentrations may show increase (or super scaling), decrease (or sub scaling), or no-change (or scaling) with cell size^31^. It is not known how the concentrations of NK cell signaling proteins considered here scales with the cell size. Therefore, we did not include any explicit model constraints to impose protein concentration homeostasis for these NK cell signaling proteins.

*(iv) cell-size control mechanisms are not included in the model (adder, sizer, timer); the choice is known to crucially alter protein dynamics across the cell-cycle so difficult to see how one can ignore the inclusion of these. See for e.g. PLoS computational biology 18.10 (2022): e1010574.*

We have revised the main manuscript (page 8) to address the above point (#(iv)), as well as the previous points (# (i)-(iii)). We have appended the text below for the convenience of the reviewer.

Mathematical models using multiple virtual cell cycle stages have been widely used to describe cell cycle kinetics^4-6^. Several of these models consider cell cycle transition rates proportional to the cell volume with a power (∝ V(t)^q^)^7^ to generate size homeostasis where the cell sizes in a dividing cell population maintains an average cell size across generations. The size homeostasis has been observed in experiments with bacterial, yeast, and some mammalian cells^8,9^. These models investigate implications of three major paradigms^8,9^ proposed to explain cell size homeostasis where cells divide after increasing their volume at birth either by a fixed amount (adder model, q=1), or when the cell size reaches a critical size (sizer model, q=∞), or after a specific time interval (timer model, q=0). The bacterial and yeast cells appear to follow a mixture of the above paradigms (e.g., sizer-like or timer-like) to implement cell size homeostasis^8^. Some of these models also consider mRNA and protein production using reduced gene regulatory models to produce homeostasis of mRNA and protein concentrations (# of mRNA/protein molecules per cell/cell volume)^7,10^. However, concentration homeostasis is usually observed for total mRNA or protein content^9^, and individual mRNA^2^ or protein^3^ concentrations may show increase (or super scaling), decrease (or sub scaling), or no-change (or scaling) with cell size^31^. Here, we focused on the effect of cell cycle transitions on NK cell signaling occurring in a substantially shorter time duration (0-256 mins, Figure 2E) compared to the NK cell division times (~ 30 hrs), thus during cell signaling, a single NK cell is less likely to undergo multiple cell divisions. Therefore, we considered a simplified model where the above cell volume-dependent transition rates or protein production rates are not explicitly considered. The effect of the cell cycle stage on protein production/degradation can be implemented as cell cycle stage-specific protein production/degradation rates.

*Minor comments: 
The literature on models of gene expression (mRNA and protein dynamics) including cell-cycle dynamics is extensive and the discussion of this paper would benefit from including more of this. Some of these papers include Biophysical Journal, 107 (2014), 301-313; Journal of theoretical biology 348 (2014): 1-11.; PLoS computational biology 12.8 (2016): e1004972; Plos one 15.1 (2020): e0226016; J. Chem. Phys. 159, 224102 (2023).*

We have added a brief discussion of the above references in our Discussion section (page 16).

Stochastic mathematical and computational models combining simple models of gene regulation and protein production and cell cycle kinetics have been developed previously to evaluate the effect of cell cycle in cell-cell variations of mRNA/proteins^11-14^, gene autoregulation^15^, and homeostasis of mRNA/protein concentrations^7,10^

*Reviewer #2 (Evidence, reproducibility and clarity (Required)): 

Summary: 
Wethington, Nayak, Jensen et al. investigated changes within protein abundances in distinct NK cell cycle stages after NKG2D stimulation of primary human NK cells and the NK cell line NKL. In addition, the authors use mathematical models to define distinct patterns of signaling protein abundances across different cell cycle stages. 
Overall, the manuscript is well written and of interest for the scientific community. However, the manuscript could benefit from additional improvements.*

Thank you for considering our manuscript to be well written and of interest for the scientific community. We have added new simulations, data analysis, and rewritten several parts of the main text to further improve the manuscript.

*Major comments 
1. It remains unclear how many replicates were used within the manuscript throughout. Please stage the number of replicates clearly. Since there is considerable variation between different human donors an n=3-5 would be preferable for the NKG2D stimulation of primary human data to draw valuable conclusions.*

Here we have used only one replicate each for the primary NK cell and NKL cell datasets for analysis of high dimensional CyTOF data. This has now been mentioned in the Results section (page 4) and caption of Figure 2A. For the primary NK cells, an additional dataset from an independent biological replicate obtained from a different donor has now been included in Supplementary Figure S10. While this provides further validation, conducting experiments on additional donors is beyond the scope of this study. We have added the corresponding text to the Results section (page 6), Discussion (page 15) and Limitations section (page 17).

To assess donor variability, we analyzed an independent biological replicate of the NKG2D-stimulated primary NK cells data generated from a different donor at multiple time points; where a small proportion (~ 6%) of measured proteins that follow non-monotonic changes and ~ 28% of proteins follow semi-monotonic changes at different time points (**Supplementary Figure S10**). However, we observed that the majority of the proteins (~ 61%) showed no change across cell cycle stages. Several proteins, including CD69, pSLP76, pNFκB, pERK, p38, and SAP, consistently exhibited semi-monotonic increases across donors, although with donor-specific timing, whereas pMAPKAPK2 and pVav1 showed non-monotonic changes at selected time points (**Figure 2E**; **Supplementary Figure S10**). Although these observations suggest conserved qualitative trends, additional biological replicates will be necessary to rigorously assess the reproducibility and generality of these patterns.

We observed differences in these patterns in primary NK cells across donors (**Supplementary Figure S10**), which could arise from donor-to-donor variations in NK cell signaling and batch effects. However, fewer than 10% of proteins still displayed non-monotonic patterns across donors.

Overall, these tests show that the variation of protein intensities across cell cycle stages can be sensitive to donor-donor variations, and the metric (e.g., geometric mean) used in quantifying the population mean. Thus, to further quantify and validate the cell cycle dependence of signaling proteins further experimentation is needed, for example, measuring CDK abundances upon NK cell stimulation and assessing their impact on biochemical signaling rates along cell cycle stages across donors would be an interesting direction for future studies. The approaches presented here serve as hypothesis generation tools for further experimentation.

*2. Did the authors compare non-reactive vs reactive NK cells after NKG2D stimulation and if yes, how does the pattern look for the signaling molecules between distinct cell cycle phases when comparing those? It would be interesting to see the distribuition of CD107a negative and positive NK cells within the different cell cycle stages upon stimulation. This would potentially also provide an internal negative control as the signaling proteins within the CD107a negative population are expected to go through less changes.*

This is an interesting suggestion. We used high and low CD107a expression to distinguish between reactive vs. non-reactive NK cell populations. We found a small (<1%) of NK cells to be a CD107a^high^ (or reactive) population for most of the time points. At t=256 min post-NKG2D stimulation, ~9.5% of the NK cell population were reactive (596 cells out of 6282 cells). We performed UMAP analysis on the reactive (596 cells) and non-reactive (5686 cells) populations and subsequently analyzed changes in protein intensities across cell cycle stages (Figure R1). We observed that for most proteins, the variation type differed between CD107a^high^ vs. CD107a^low^ populations. However, given the small size of the sample for the reactive cell population, the separation of the cell cycle stages using UMAP could be error prone, thus we decided to not include this result in the manuscript.

**Figure R1.** **Variation of protein intensities in reactive and non-reactive populations of NKG2D-stimulated primary CD56^dim^ NK cells at 256 minutes post-stimulation presented in Figure 2E (main text).** The variations of the mean log_e_ (Intensity) of signaling proteins across cell cycle transitions, G1→ S, and S → G2-M, are shown in terms of nine different types of variations. A change (increase or decrease) is marked when the change in the mean log_e_ (Intensity) from G1→ S, or S→G2-M, has a statistical significance level α = 0.05 in one sided t-test. The nine types are characterized by the changes in (G1→ S, S → G2-M) as follows: (increase, increase), (increase, no-change), (no-change, increase), (increase, decrease), (decrease, increase), (decrease, no-change), (no-change, decrease), (decrease, decrease), (no-change, no-change).

*3. The link between the first part (NKG2D stimulation) and second part (mathematical modeling) remains a bit unclear. Was any of the NKG2D stimulation data used to train the mathematical modeling? If not a potential way to improve the link would be to describe the mathematical modeling first and subsequently validate certain patterns in the NKG2D modeling or to compare cytokine only induced changes (only IL-2) to receptor signaling changes (NKG2D stimulation).*

We thank the reviewer for the suggestion. We have now used the CyTOF data to train our mathematical model. Reviewer #1 had a similar comment. To address the comment, we used experimentally observed pS6 intensities at 4 min and pAkt intensities at 8 min across cell cycle stages to train our in-silico model and tested whether the model with neutral or the active case describes the signaling kinetics more appropriately. Comparison of the two cases using the Akaike Information Criterion (AIC) shows that the active case better explains the variations of protein abundances including the monotonic rise across cell cycle stages for these two proteins. Please refer to the revised manuscript (Results section, page 15), revised Figures 4D-4E, and our response to Reviewer #1 (Response #1) for more details.

Next, we evaluated whether the protein signaling kinetics in NK cells as measured in our CyTOF experiments are described more preferably by active or neutral case. We chose two types of signaling kinetic patterns: (1) average pS6 shows a non-monotonic behavior; a decrease for G1→ S followed by an increase for S→ G2-M at t=4 min post-NKG2D stimulation and (2) average pAkt increases monotonically from G1→ S→ G2-M at t=8 min post-NKG2D stimulation. We used a non-linear enzymatic phosphorylation model (**Figure 4A**) for pS6 and pAkt, where the phosphorylation rate, r_p_, is the same (neutral case) or can vary (active case) across cell cycle stages. The model is simulated for a small sub-volume (~ 0.132 μm^3^, Supplementary Table S1) within the cell where the reactants can be well mixed. Using the distributions of pS6 and pAkt measured in CyTOF at t=2 min as initial distributions of the number of protein molecules for the product (A*) within the sub-volume, we evolved the abundances of pS6 and pAkt in individual cells until t=4 min and t=8 min, respectively, using the model for the neutral and active cases (see Methods). The measured abundances of pS6 at 4 min and pAkt at 8 min in the CyTOF experiments were used to estimate the rate constant(s) (r_p_ in the neutral case, and three cell cycle stage-dependent r_p_ values in the active case) by minimizing the residual sum of squares (RSS) between the model and the data for the arithmetic mean of the log of the abundances (or the log of the geometric mean). Using Akaike Information Criterion (AIC)^1^ (see Methods), an approach for model selection, we found that the active scenario described the measured data at t=4 min and 8 min better (ΔAIC = -32 for pS6 and -47 for pAkt) compared to the neutral scenario for both signaling proteins. Therefore, both for monotonic and non-monotonic changes across cell cycle stages, the cell cycle-dependent phosphorylation of these signaling proteins is more consistent with the data.

*Minor comments: 
1. The level of NKG2D is not shown within manuscript and could be added as an additional supplementary figure.*

We now have added NKG2D expression in the top row of Supplementary Figure S2A.

*2. The authors mention CDKs influencing cell signaling. Did the authors track the abundance of CDK molecules upon NK cell stimulation?*

This is an interesting suggestion. We were unable to measure CDK abundance in our CyTOF experiments, as appropriately labeled antibodies for these proteins were not available at the time. This represents a potential direction for future studies. We have added the following text in the Discussion (page 17).

Thus, to further quantify and validate the cell cycle dependence of signaling proteins further experimentation is needed, for example, measuring CDK abundances upon NK cell stimulation and assessing their impact on biochemical signaling rates along cell cycle stages across donors would be an interesting direction for future studies.

*3. Figure 2E shows a lot of information and is a bit crowded. Potentially it would be easier to split the information up? Show a heatmap of the expression of the significant proteins at all different timepoints and then show the abundance changes in detail for a few proteins for specific timepoints.*

Now we have divided the heatmap in Figure 2E into two groups – (a) surface markers and (b) intracellular markers. The expressions of key NKG2D signaling proteins across cell cycle stages are shown in Fig. 2G and Supplementary Figure S2A.

*Reviewer #2 (Significance (Required)):* 

*General assessment:* 
*The manuscript provides an interesting mathematical modeling as well as CyTOF data from NK cell stimulations about differences in protein abundances throughout different cell cycle stages of NK cells. The data of the NK cell stimulation could be better linked to the mathematical modeling to make a stronger case for the robustness of the model and for more mechanistic conclusions. The manuscript contains a lot of data which is sometimes presented to condensed (Figure 2), the manuscript could benefit from a clearer red line throughout/focus on key molecules.*

We appreciate the positive remark. We have major revisions to the manuscript to address the above comments. We hope the reviewer will be satisfied with the revised manuscript.

*Audience: 
The data presented is of interest for the specialized NK cell community, but the discussion section could be improved by making a stronger case of how the herein presented data/model will benefit further studies within the NK cell or general immunology field. 

My field of expertise: NK cell biology, tissue-resident NK cells*.

*Reviewer #3 (Evidence, reproducibility and clarity (Required)):* 

*Summary* 
*In this study, Weethington et al investigate how the abundance/activity of signaling proteins change over time following stimulation of NK cells and if the dynamics of these changes are coupled to cell cycle progression. Using CyTOF to measure these proteins in single cells and using several NK cell models, the investigators categorize proteins by the dynamics of these changes as cells progress through G1, S, and G2/M. The investigators indicate that the majority of proteins increase monotonically or semi-monotonically during cell cycle progression, while others exhibit non-monotonic changes - increasing from G1 -> S and then decreasing form S -> G2/M or vice-versa. The authors then use these data to inform mathematical models to identify the cellular processes that may give rise to these non-monotonic changes, identifying protein synthesis, degradation, or signaling kinetics as potential mechanisms.* 

*Major comments* 
*1. I do not understand the rationale for comparing time points (post-stimulation) between progressive cell cycle phases. Although there is a fixed temporal ordering to cell cycle phases (G1 -> S -> G2/M), there is no temporal relationship between protein abundance measurements at a post-stimulation time point in different cell cycle phases. For example, take CD69 in Fig 2E,G: the authors cite non-monotonous changes occurring at the 32, 64, and 256 min timepoints and semi-monotonic changes at all other time points. The abundance of CD69 at 32 min post-stimulation in G1 has no temporal relationship to the 32 min time point in S or G2 phase, so it is not clear how a stagement about monotonicity can be made in this context?*

*I believe the appropriate analysis strategy to interrogate the question posed by the authors in this paper is to compare the entire time-course of protein abundance between phases (i.e. the shape/magnitude of change in protein abundance in G1 vs S vs G2). Through this lens, the CD69 data in Fig 2G would suggest that the decrease in protein abundance at later time points (relative to untreated within the same phase) is larger in S phase than in G1 or G2.*

We thank the reviewer for this insightful comment. The duration (~ 256 mins) of NKG2D signaling kinetics probed here is several times shorter than the duration of cell cycle transitions for primary NK cells, which takes approximately 30 hours to divide. Thus, we expect very few cells to undergo cell cycle transitions during NKG2D signaling. Nevertheless, the qualitative nature of longitudinal changes in average expressions of signaling proteins in cells in different cell cycle stages are similar, as the NK cells in different cell cycle stages are being stimulated with the NKG2D antibodies (Supplementary Figure S11). For example, as time progresses some of signaling products such as CD107a builds up in these NK cells. This is reflected in the rise in the population averages of CD107a with time. In contrast, the mean CD69 expression stays the same until t=16 mins for NK cells across cell cycle stages, with consistently higher mean values in the G2-M stage compared to the G1 and S phases. The observed monotonic or non-monotonic changes in expressions of the signaling proteins across cell cycle stages at a fixed time point thus reflect how NK cells in the cell stages have responded to the NKG2D receptor stimulation until that time point, which we conclude arise due to the differences in the abundances of the enzymes and the substrates pertaining to the signaling kinetics in the cell cycle stages and any direct inference of cell cycle driving proteins such as CDK. We have discussed these points in the revised manuscript and shown the kinetics of the mean abundances of some of the signaling proteins in the supplementary material (Supplementary Figure S11). Furthermore, we have used our model to further quantify the mechanisms involving cell cycle regulation of the signaling kinetics. Please refer to our response #1 to Reviewer #1. We now have added the following text in the Discussion (page 15).

We further examined the time courses of average protein intensities across cell-cycle stages (**Supplementary Figure S11**). This analysis indicates that some proteins, such as CD107a, increase over the duration of NKG2D stimulation, whereas others, such as pCrkL and NKG2D decrease with time, while many proteins did not display such simple trends in their temporal changes.

*2. It should also be noted that the CD69 dynamics following stimulation is completely different in primary cells (Fig 2) vs the NK cell line (Fig S3), making interpretation and generalization very difficult.*

NKL is an engineered, transformed cell line and may not represent the healthy primary NK cells. There could be altered signaling pathway. In our analysis, we observed that CD69 dynamics differ between primary NK and NKL cells, except at 256 min post-stimulation, where both show non-monotonic behavior. We have now added a biological replicate (a different donor) of NKG2D-stimulated primary NK cell data in Supplementary Figure S10. Corresponding text has been added to the Results section (page 6) and Discussion (page 15). For further details, please see Comment #1 by Reviewer #2.

To assess donor variability, we analyzed an independent biological replicate of the NKG2D-stimulated primary NK cells data generated from a different donor at multiple time points; where a small proportion (~ 6%) of measured proteins that follow non-monotonic changes and ~ 28% of proteins follow semi-monotonic changes at different time points (Supplementary Figure S10). However, we observed that the majority of the proteins (~ 61%) showed no change across cell cycle stages. Several proteins, including CD69, pSLP76, pNFκB, pERK, p38, and SAP, consistently exhibited semi-monotonic increases across donors, although with donor-specific timing, whereas pMAPKAPK2 and pVav1 showed non-monotonic changes at selected time points (**Figure 2E**; **Supplementary Figure S10**). Although these observations suggest conserved qualitative trends, additional biological replicates will be necessary to rigorously assess the reproducibility and generality of these patterns.

We observed differences in these patterns in primary NK cells across donors (**Supplementary Figure S10**), which could arise from donor-to-donor variations in NK cell signaling and batch effects. However, fewer than 10% of proteins still displayed non-monotonic patterns across donors.

*3. It is also difficult to assess the magnitude of differences in protein abundance given that there are often no measures of variance indicated in the bar plots visualizing these changes (e.g. Fig 2G, Fig S2B).*

We have now added error bars to the bar plots in Fig. 2G of the main manuscript and Supplementary Figure S2A, as suggested by the reviewer.

*4. I am aware that the authors use a pair of one-sided t tests to make stagements of statistical significance for these comparisons. However, in single-cell assays of this scale with hundreds to thousands of data points per condition, t tests are prone to Type I error and often overpowered to identify truly meaningful differences. Is a >5% decrease in mean abundance from G1 to S phase in a single experiment (independent replicates do not appear to have been performed) and no follow-up validation experiments sufficient to make the stagement that this decrease is biologically meaningful? And then stratify proteins into classes based on these relatively small changes?*

We agree that the large number of cells in single-cell datasets can yield statistically significant differences in mean protein intensities between consecutive cell cycle stages (e.g., G1 and S phases) and such statistical significance should be interpreted with caution given variations across biological replicates. Reviewer #2 also had a similar comment (see Comment#1 for Rev. #2). We have taken two additional steps to address the above question.

(a) We carried out our analysis on an additional biological replicate from a different donor to find that the percentage of signaling proteins showing non-monotonic changes is <10% for all the donors; however, the proteins and the time intervals where they occur are different across donors. This is expected as NK cell responses show large variations across donors, and the measurements could also be affected by batch effects. We discuss donor-donor variations of our results in the main text (page 6 in the Results section, page 15 in the Discussion) and Figure S10 in the supplementary material.

To assess donor variability, we analyzed an independent biological replicate of the NKG2D-stimulated primary NK cells data generated from a different donor at multiple time points; where a small proportion (~ 6%) of measured proteins that follow non-monotonic changes and ~ 28% of proteins follow semi-monotonic changes at different time points (Supplementary Figure S10). However, we observed that the majority of the proteins (~ 61%) showed no change across cell cycle stages. Several proteins, including CD69, pSLP76, pNFκB, pERK, p38, and SAP, consistently exhibited semi-monotonic increases across donors, although with donor-specific timing, whereas pMAPKAPK2 and pVav1 showed non-monotonic changes at selected time points (**Figure 2E**; **Supplementary Figure S10**). Although these observations suggest conserved qualitative trends, additional biological replicates will be necessary to rigorously assess the reproducibility and generality of these patterns.

We observed differences in these patterns in primary NK cells across donors (**Supplementary Figure S10**), which could arise from donor-to-donor variations in NK cell signaling and batch effects. However, fewer than 10% of proteins still displayed non-monotonic patterns across donors.

(b) We performed a more general non-parametric calculation of the p-values for the changes in the CyTOF intensities using a different mean (shifted geometric mean). These tests reveal (Supplementary Fig. S12) that some of the variations are sensitive to the metric (e.g., shifted geometric mean) used for quantifying the mean values, though many of the proteins showed similar variation patterns between Figure 2E and Supplementary Figure S12. We described details regarding these tests in the Results (page 5) and Methods (page 19) in main text.

To examine the robustness of the protein abundance variation patterns presented in **Figure 2E**, we additionally applied a non-parametric statistical test and computed the changes in protein abundances using shifted geometric mean (Methods and **Supplementary Figure S12**). The shifted geometric mean-based analysis recapitulated trends similar to those observed in Figure 2E. For example, proteins including pAkt, pMAPKAPK2, pNFκB, pPLK1, pS6, and pVav1, which exhibited non-monotonic behavior based on the geometric mean, also showed non-monotonic behavior when analyzed using the shifted geometric mean.

**Testing protein variation patterns using the non-parametric test**

To verify the robustness of the results presented in **Figure 2E,** which were obtained using a parametric *t*-test, we further examined the variations of protein intensity patterns across cell cycle stages in CD56^dim^ NKG2D-stimulated primary NK cells using a non-parametric approach based on shifted geometric mean. For this method, the null hypothesis is that the true means of the two intensity distributions are equal (H_0_: μ_1_=μ_2_), where μ_1_, μ_2_ represent the means of the two distributions. To estimate the p-values, we bootstrapped both the observed distributions n_b_ times to calculate the difference between bootstrapped means (Δ=μ_1_^*^- μ_2_^*^), where μ_1_^*^, μ_2_^*^ denote the means computed from protein intensities $x_{i}^{*}$ in bootstrapped samples 1 and 2, respectively. Finally we calculated p values from the distribution of Δ using the formula^16^, p-value = $2 \times\min\left( p,1-p \right),$ where p= number of (Δ > 0) / n_b_.

*Shifted geometric mean*: To calculate the shifted geometric mean in bootstrapped samples (**Supplementary Figure S12**), we used $\mu^{*}=\exp\left( \frac{1}{n}\sum_{i} log(x_{i}^{*}+c) \right)-c$, where c is a small positive constant, which was set to 10^-11^. This calculation was applied to both μ_1_^*^ and μ_2_^*^.

We have mentioned the following in the limitation section (page 17).

Overall, these tests show that the variation of protein intensities across cell cycle stages can be sensitive to donor-donor variations, and the metric (e.g., geometric mean) used in quantifying the population mean. Thus, to further quantify and validate the cell cycle dependence of signaling proteins further experimentation is needed, for example, measuring CDK abundances upon NK cell stimulation and assessing their impact on biochemical signaling rates along cell cycle stages across donors would be an interesting direction for future studies. The approaches presented here serve as hypothesis generation tools for further experimentation.

*Reviewer #3 (Significance (Required)): 

Significance 
Our current knowledge of the mammalian cell cycle comes mostly studies in epithelial and fibroblast cells. A better understanding of the cell cycles of other cell types, how it is regulated, and how it influences other cell biological events would be a significant benefit to the field*

We appreciate the positive remark.

*General assessment: I believe that this study has fundamental concerns (described above) that must be addressed before this manuscript should advance to publication 
Audience: Basic research, cell cycle and immunology audiences.*

We have made major revisions in the manuscript to address the reviewers’ comments. We hope the revised manuscript will be satisfactory to the reviewer.

*My background is in experimental and computational cell cycle biology*

1	Akaike, H. in *International encyclopedia of statistical science*	41-42 (Springer, 2025).

2	Chen, Y., Zhao, G., Zahumensky, J., Honey, S. & Futcher, B. Differential scaling of gene expression with cell size may explain size control in budding yeast. *Molecular cell* **78**, 359-370. e356 (2020).

3	Lanz, M. C. *et al.* Increasing cell size remodels the proteome and promotes senescence. *Molecular cell* **82**, 3255-3269. e3258 (2022).

4	Vittadello, S. T., McCue, S. W., Gunasingh, G., Haass, N. K. & Simpson, M. J. Mathematical models incorporating a multi-stage cell cycle replicate normally-hidden inherent synchronization in cell proliferation. *Journal of the Royal Society Interface* **16**, 20190382 (2019).

5	Yates, C. A., Ford, M. J. & Mort, R. L. A multi-stage representation of cell proliferation as a Markov process. *Bulletin of mathematical biology* **79**, 2905-2928 (2017).

6	Perez-Carrasco, R., Beentjes, C. & Grima, R. Effects of cell cycle variability on lineage and population measurements of messenger RNA abundance. *Journal of the Royal Society Interface* **17**, 20200360 (2020).

7	Jia, C., Singh, A. & Grima, R. Concentration fluctuations in growing and dividing cells: Insights into the emergence of concentration homeostasis. *PLoS computational biology* **18**, e1010574 (2022).

8	Jun, S. & Taheri-Araghi, S. Cell-size maintenance: universal strategy revealed. *Trends in microbiology* **23**, 4-6 (2015).

9	Xie, S., Swaffer, M. & Skotheim, J. M. Eukaryotic cell size control and its relation to biosynthesis and senescence. *Annual review of cell and developmental biology* **38**, 291-319 (2022).

10	Lin, J. & Amir, A. Homeostasis of protein and mRNA concentrations in growing cells. *Nature communications* **9**, 4496 (2018).

11	Schwabe, A. & Bruggeman, F. J. Contributions of cell growth and biochemical reactions to nongenetic variability of cells. *Biophysical journal* **107**, 301-313 (2014).

12	Soltani, M., Vargas-Garcia, C. A., Antunes, D. & Singh, A. Intercellular variability in protein levels from stochastic expression and noisy cell cycle processes. *PLoS computational biology* **12**, e1004972 (2016).

13	Wang, Y., Yu, Z., Grima, R. & Cao, Z. Exact solution of a three-stage model of stochastic gene expression including cell-cycle dynamics. *The Journal of Chemical Physics* **159** (2023).

14	Dessalles, R., Fromion, V. & Robert, P. Models of protein production along the cell cycle: An investigation of possible sources of noise. *Plos one* **15**, e0226016 (2020).

15	Gomez, D., Marathe, R., Bierbaum, V. & Klumpp, S. Modeling stochastic gene expression in growing cells. *Journal of theoretical biology* **348**, 1-11 (2014).

16	Tibshirani, R. J. & Efron, B. An introduction to the bootstrap. *Monographs on statistics and applied probability* **57**, 1-436 (1993).
