# Supplementary material for: Natural killer cell receptor signaling and activation depend on cell cycle stages
Source: PLoS Comput Biol. 2026 Oct 13;22(10):e1014825. doi: 10.1371/journal.pcbi.1014825 (PMC13705607; doi:10.1371/journal.pcbi.1014825)
Supplement: Supplementary file 10 [file pcbi.1014825.s021.docx]

Dear Editor,

Thank you for giving us the opportunity to revise our manuscript. We are glad that Reviewer #1 and #3 were satisfied with the revised manuscript. Reviewer #2 had a single sentence comment for the authors, “*This revision still does not resolve my issue with the tonicity analysis*.” We revised the main text and added new figures in the main text (revised Fig. 2) and the supplementary material (Supplementary Figures 2-4) in the revised version to address the reviewers’ comments. The one-line non-specific comment does not provide much guideline for further revising the manuscript. However, we edited to main text and revised Fig. 2G to provide some context for analyzing patterns of protein abundances across cell cycle stages at any time point (referred as the “tonicity” analysis by the reviewer). This type of analysis of patterns of protein levels across cell cycles stages has been used previously in phospho-proteomic studies investigating protein phosphorylation in cell cycle (PMID: 29228002, 32051232). We have mentioned this in the current revision.

We hope you will find the current version suitable for publication in PLOS Computational Biology.

Sincerely,

Lewis L Lanier

Jayajit Das

---------------------------------- Point-by-point response to reviewers comments--------------------

**Reviewers' comments:**

Reviewer's Responses to Questions

Reviewer #2

*This revision still does not resolve my issue with the tonicity analysis*.

We were unclear which part of the revision was still an issue for the reviewer. We believe we made major changes in the main text, main text figures (Fig. 2) and supplementary figures (Supplementary Figures 2-4) to address the reviewers’ comments on “tonicity” analysis. Since, the above comment does not provide any specific suggestion, we revised the main text to provide some context regarding our analysis. Similar analysis has been used previously to analyze cell cycle dependencies of phosphorylation in phospho-proteomic studies (PMID: 29228002, 32051232), which is noted in the current version. We have further revised our schematic figure (Fig. 2G) to provide connection between such snapshot analysis and signaling kinetics. Note, we also showed longitudinal data for each cell cycle stage for the protein abundances in the revised manuscript as the reviewer suggested.
