# Supplementary material for: Natural killer cell receptor signaling and activation depend on cell cycle stages
Source: PLoS Comput Biol. 2026 Oct 13;22(10):e1014825. doi: 10.1371/journal.pcbi.1014825 (PMC13705607; doi:10.1371/journal.pcbi.1014825)
Supplement: Supplementary file 11 [file pcbi.1014825.s022.docx]

Dear Dr. Wodarz,

Thank you for giving us the opportunity to revise our manuscript. We have revised the main text and Figure 2 in the main text and the supplementary material (Supplementary Figures 3-4, 7, 10, and 12) to address the reviewers’ comments. A detailed response to your comment is appended below.

We hope you will find the current version suitable for publication in PLOS Computational Biology.

Sincerely,

Lewis L Lanier

Jayajit Das

---------------------------------- Point-by-point response to reviewers comments--------------------

**Editors' comments:**

*Thank you for your careful revision of the manuscript. The revised version has addressed many of the concerns raised during review, and I believe the manuscript can be considered further pending a small number of focused changes.*

*Having looked through the previous comments by reviewer 2, and your revisions in response, my understanding is the following: The main remaining issue concerns the analysis of protein abundance patterns across cell-cycle stages at fixed post-stimulation time points. The revised manuscript now more clearly distinguishes these cross-sectional “snapshot” comparisons from the longitudinal signaling kinetics within each cell-cycle stage, which substantially improves the interpretation. However, the concern regarding the longest stimulation time remains important: at 256 min, cells may have transitioned between cell-cycle stages during the treatment interval, so their stage at fixation may not reflect their stage at the start of stimulation. The manuscript now acknowledges this explicitly.*

*If I understand this correctly, I would suggest to remove the 256-min time point from the monotonic/non-monotonic snapshot analysis and the associated summary percentages, or provide an analysis demonstrating that the main conclusions are unchanged when the analysis is restricted to time points of 64 min or less. In addition, please ensure throughout the manuscript that the G1/S/G2+M comparisons at fixed post-stimulation times are described strictly as cross-sectional comparisons and are not interpreted as temporal progression of the same cells through the cell cycle.*

We thank the editor for carefully considering our responses to the points raised by Reviewer#2. We have removed the time points > 64 mins from all the monotonic/non-monotonic snapshot analysis presented in the manuscript figures (Figure 2H in the main text, and Figures S3B, S4B, S7, S10 and S12 in the supplementary material) and revised the associated summary percentages. The cross-sectional analyses are not connected with any time progression analyses in the revised manuscript.
